# A computational framework for stability-constrained offline PI tuning of BLDC FOC systems using gray wolf optimization

**DOI:** 10.1016/j.mex.2026.104114

**Published:** 2026-08-22

**Authors:** Rakhmat Agung Ramadhani, Ika Noer Syamsiana, Arwin Datumaya Wahyudi Sumari, Moechammad Sarosa

**Affiliations:** a Department of Electrical Engineering, State Polytechnic of Malang, Malang, East Java, 65141, Indonesia; b Office of Special High-Rank Officer, Indonesian Air Force Headquarters, Jakarta Timur, DKI Jakarta, 13870, Indonesia

**Keywords:** BLDC, Field-oriented control, Gray wolf optimization, PI controller

## Abstract

Proportional-Integral (PI) controllers are widely used in Field-Oriented Control (FOC) systems for Brushless DC (BLDC) motors because they are simple. However, their performance relies heavily on proper parameter tuning. Traditional methods like Ziegler-Nichols or trial-and-error can give unpredictable results when operating conditions or loads change. This study introduces a computer-based approach for stable offline PI tuning in BLDC FOC systems using the Gray Wolf Optimization (GWO) algorithm.

• The proposed framework integrates mathematical modeling of the BLDC motor, stability rules, multi-objective evaluation, and automated optimization steps into a single software process.

• The proposed framework also offers repeatable tuning steps, methods for setting initial parameters, progress tracking, and performance checks, making it easier to reuse in engineering projects.

• The results show that this method significantly improves system response. With no load, speed overshoot dropped from about 20% (0.96 pu) in standard FOC to around 3% (0.82 pu) with FOC-GWO, and the initial torque surge decreased from 0.118 pu to 0.072 pu. When a load disturbance occurs, the FOC-GWO system has a smaller speed drop, recovers faster, and shows less fluctuation in speed and torque than the traditional method.

## Graphical abstract

**Figure:**
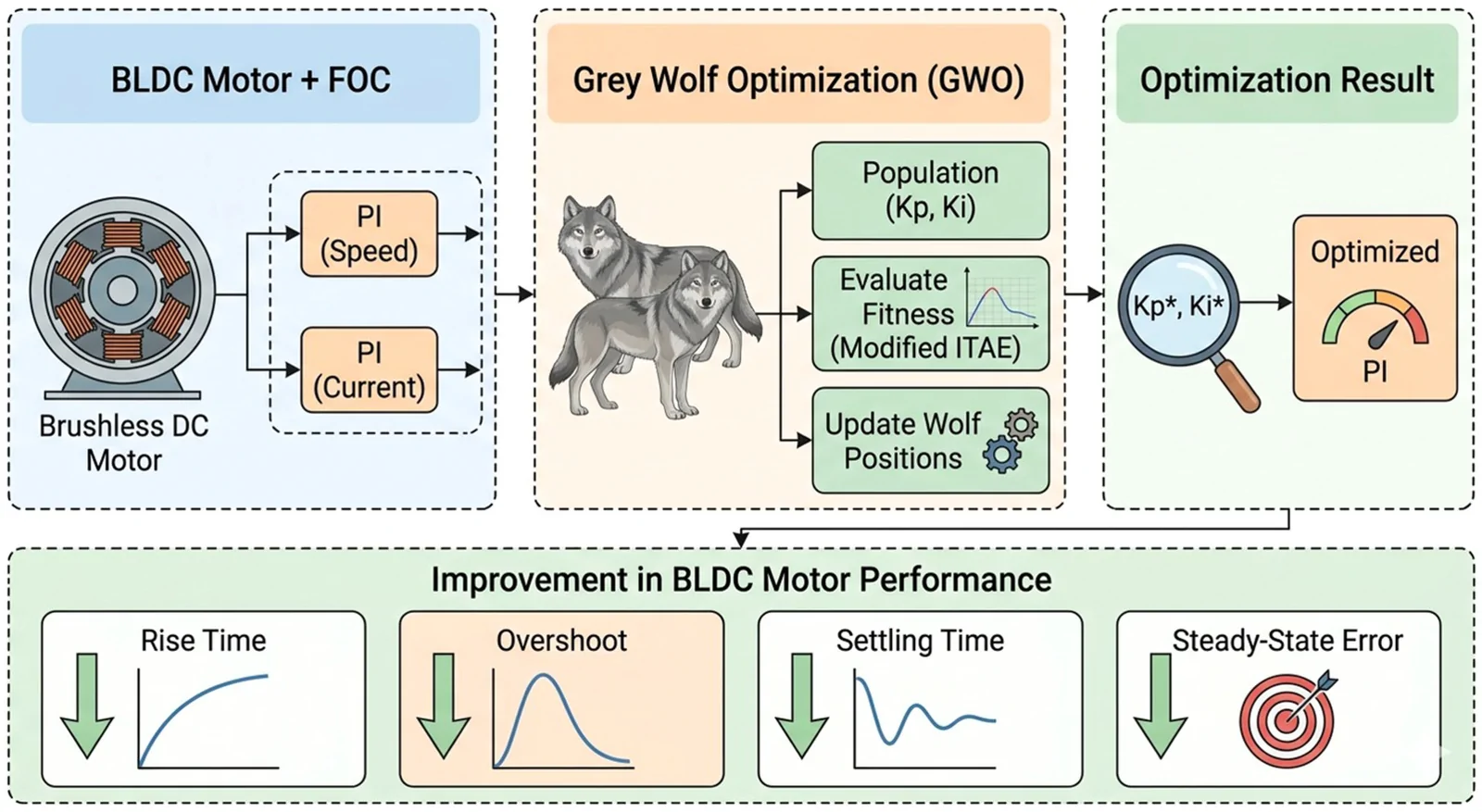

## Specifications table

**Table:** 

**Subject area**	Computer Science
**More specific subject area**	Improved performance of motor control system
**Name of your method**	Optimal PI Parameter Tuning Method for FOC Using GWO to Improve Motor Performance
**Name and reference of original method**	N/A
**Resource availability**	https://www.mathworks.com/help/ti-c2000/ug/foc-hall-sensor-example.html The complete MATLAB implementation of the proposed GWO-based PI tuning framework is publicly available at: https://github.com/RakhmatAgung/Grey-Wolf-Optimization-for-PI-Parameter-Tuning/tree/main/GWO%20Simulation

## Background

The Proportional-Integral (PI) controller is one of the most widely used methods in control systems due to its simple structure, ease of implementation, and minimal computational requirements [1,2]. In Permanent Magnet Motor drive systems with Field-Oriented Control (FOC), the PI controller is also used in the speed loop to ensure that the motor speed follows the reference and remains stable during disturbances [[3], [4], [5], [6]]. Although PI controllers have been widely implemented, their performance is highly influenced by the selection of PI parameters that match the motor characteristics and operating conditions [4,7,8].

Some conventional tuning methods, such as Ziegler-Nichols, symmetric optimization, and frequency domain techniques were primarily developed for simple linear systems and do not directly account for parameter changes, load disturbances, or multiple performance objectives [[9], [10], [11], [12]]. In FOC systems, which involve interactions among current, flux, and speed variables, operating conditions can change [13], so parameters derived from general rules do not always yield consistent responses. Another commonly used approach is the trial and error method [14,15]. This involves trying various parameter combinations until a suitable response is obtained. However, the results are highly dependent on the engineers’ experience and involve a repetitive process. In systems with many parameters and nonlinear relationships, this process can become less systematic and difficult to replicate [14,16,17].

To reduce reliance on manual tuning, metaheuristic optimization techniques have been widely used to determine PI controller parameters [[18], [19], [20]]. Population-based algorithms enable a more structured search for parameters and can handle nonlinear objective functions and objective functions with constraints [[21], [22], [23]]. One algorithm worth considering is the Grey Wolf Optimization (GWO), which employs a population-based search mechanism that balances exploration and exploitation of the solution space. This algorithm does not require derivative information and is relatively easy to implement.

However, in several reviewed studies, there remains a gap where metaheuristic-based PI tuning focuses solely on minimizing the error value of the objective function used without considering the varying mechanical dynamics of the motor and the resulting convergence quality [[24], [25], [26], [27]]. This often results in parameters that appear optimal in terms of error values but actually operate too close to the instability threshold or lack robustness when implemented on actual hardware.

Given these limitations, this paper proposes a structured offline PI tuning framework for the speed loop in FOC systems. In this approach, candidate parameters generated by GWO are evaluated with respect to stability constraints prior to the final performance evaluation. This screening process is integrated into the optimization stages, ensuring that only parameters that meet the system’s dynamic requirements are considered during the search. Furthermore, the objective function is modified by adding overshoot and energy penalties to force the algorithm to find the optimal trade-off between speed response performance (from the ITAE), mechanical safety (from the overshoot penalty), and hardware feasibility/power efficiency (from the energy penalty). Thus, it is expected that the obtained PI parameters will be significantly more robust when deployed to a real motor control system.

Essentially, GWO has been widely used for tuning PI parameters in various control applications [[28], [29], [30]]. However, in this study, the main contribution lies not in the optimization algorithm itself, but in the addition of three integrated elements that extend its application to PI tuning that takes stability aspects into account for FOC-based BLDC drives:

1) Modified objective function that complements the conventional ITAE-based tracking error with explicit overshoot and control energy penalty terms,
2) Boundary constraint mechanism that relocates out-of-range PI gains to the midpoint between their previous position and the violated bound, rather than clipping directly to the boundary as in conventional GWO tuning, this preserves population diversity and reduces the premature convergence that direct clipping can cause when multiple agents cluster at the search-space limits,
3) Automated and reproducible offline tuning workflow that links the objective function with these constraints to the FOC plant model and generates validated gain values for direct implementation on hardware. Overall, these elements distinguish the proposed framework from general GWO applications for PI tuning.

## Method details

The offline automatic tuning procedure is fully implemented within the MATLAB programming environment, and the resulting PI parameters will be directly implemented using the TMS320F28035 Digital Signal Processor (DSP) board to obtain real-time data on hardware operation, the response of which will then be analyzed. The tuning method utilizes the Grey Wolf Optimizer (GWO) metaheuristic algorithm to systematically search for and determine the optimal proportional (Kp) and integral (Ki) gains for the outer speed control loop of a Brushless DC (BLDC) motor system driven by FOC. The algorithm’s execution is divided into four interconnected functional modules to ensure computational transparency and ease of replication:

• **Core Optimization Module:** Coordinates the overall initialization of GWO, the parameter exploration loop, and the alpha agent tracking metric.
• **Plant Configuration Module:** Summarizes the complete hardware constraints, measurable parameters, and electrical/mechanical dynamics of the target BLDC motor.
• **Objective Function Evaluation Module:** Functions as a numerical solver that integrates candidate controller gains into the system’s dynamic differential equations, solves for transient responses, and calculates quantitative fitness scores.
• **Constraint Module:** Monitors and dynamically constrains candidate control variables within strict operating limits to ensure numerical stability during the search process.

**Fig. 1:**
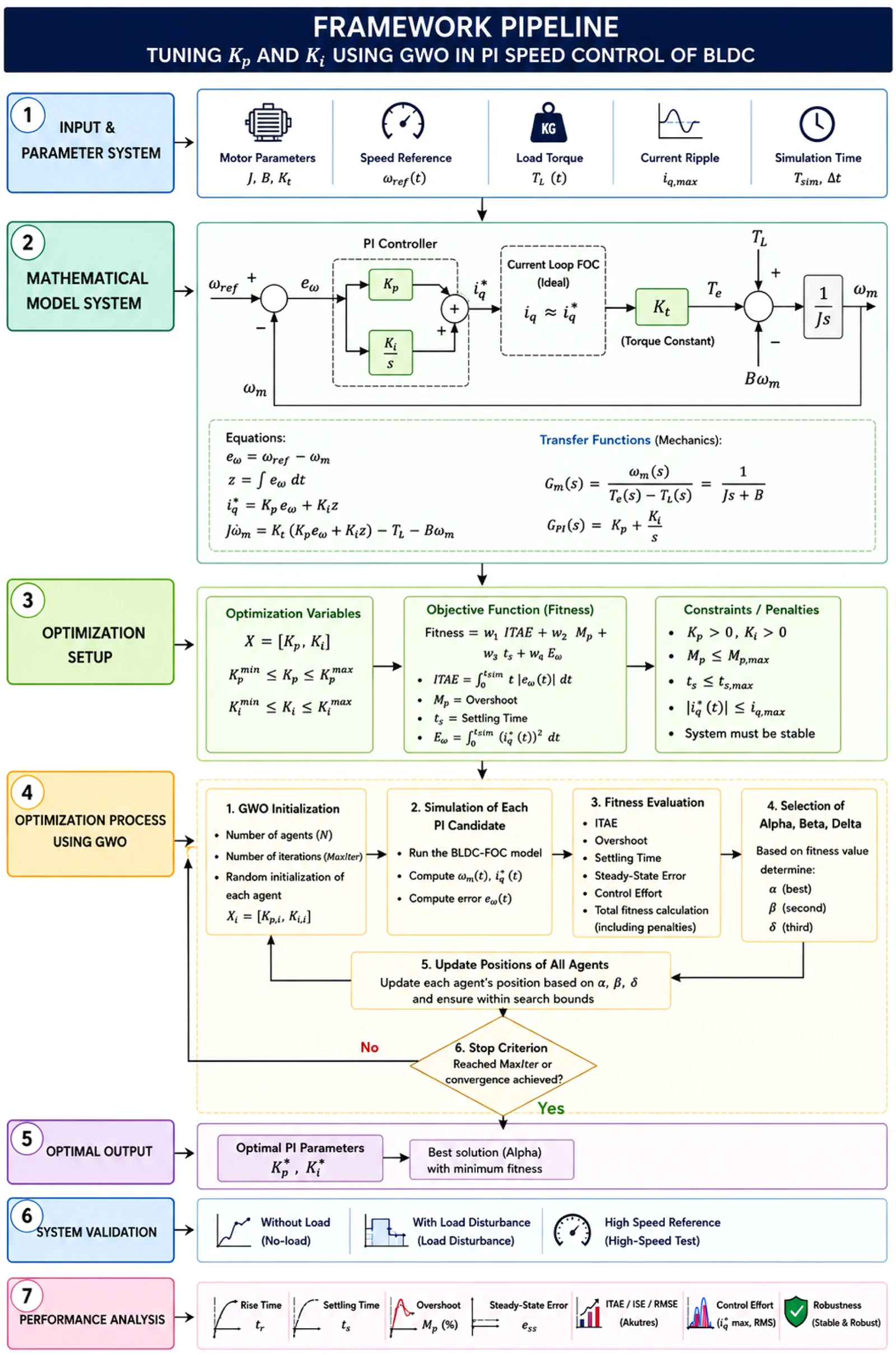
Framework pipeline of the proposed method.

However, before performing the tuning process using GWO, it is necessary to first understand how the FOC scheme works, the mathematical model of the BLDC motor, and the integration of the two to ensure the reproducibility of this study. The workflow framework is shown in Fig. 1.

## Field-Oriented control scheme

**Fig. 2:**
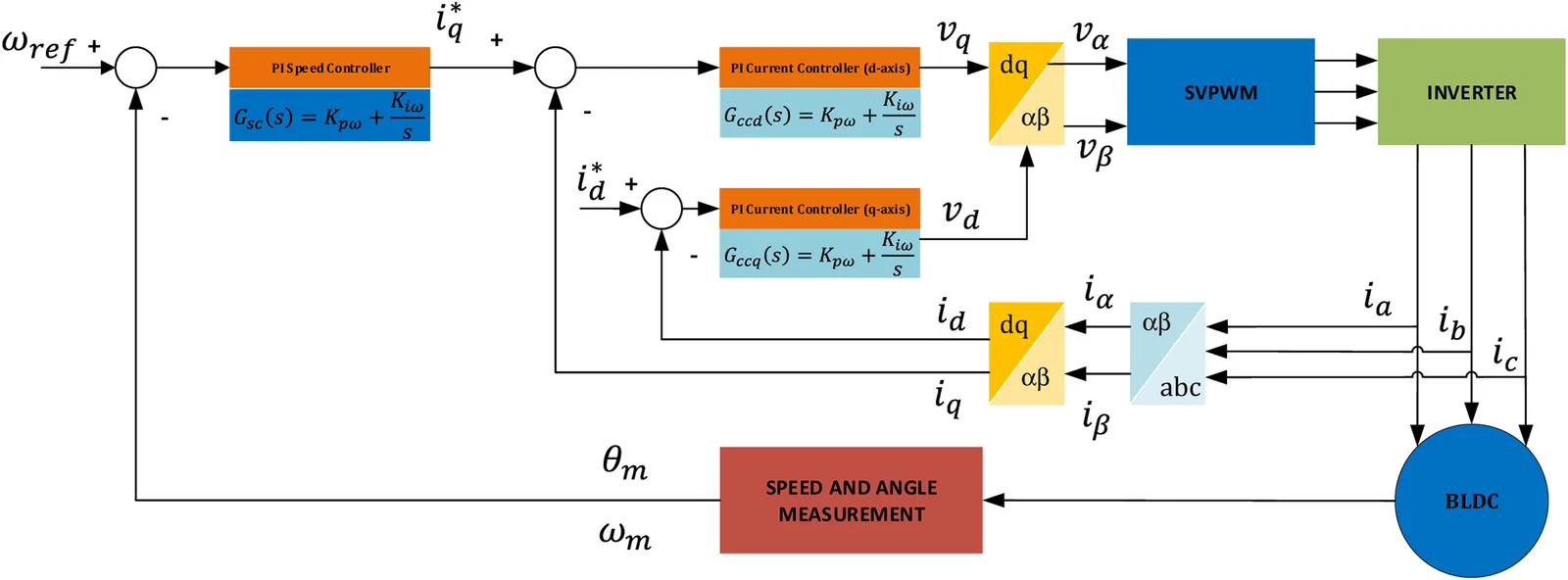
Field-oriented control block scheme.

The conventional FOC block diagram is shown in Fig. 1. The speed and angle estimators estimate the angle and speed from feedback from the position and speed sensors. The purpose of the Clark block is to obtain the three-phase current values. In the Park block, the three-phase current is then converted to dq components. The actual speed is compared with the reference speed. The error value is then sent to the PI controller in the Iq Current Control block. The goal is to generate a torque command based on the speed difference. Next, the Id reference is set to zero to achieve maximum torque. The Iq and Id references are converted into the $V_{abc}$ reference via the inverse Park transformation. The SVPWM block converts this into a vector space PWM signal, which is ultimately fed to the current controlled inverter to generate the three-phase voltage required to power the motor shown in Fig. 2.

A three-phase motor operates using three alternating currents ($I_{a},I_{b},I_{c}$) that are spatially separated by 120° Monitoring and controlling three variables that constantly change over time is certainly very complex. This is where the Clarke transformation serves as an initial simplification. This transformation converts the three-phase coordinate system (a, b, c) into a static two-phase coordinate system with mutually perpendicular (90°) axes, which we refer to as the α and β axes. Here is the transformation equation:

(1) $$\left\{ \begin{array}{c} i_{a}i_{b}i_{c}=0\\ i_{\alpha }=\frac{2}{3}(i_{\alpha }-\frac{1}{2}i_{b}-\frac{1}{2}i_{c}\\ i_{\beta }=\frac{2}{3}(\frac{\sqrt{3}}{2}i_{b}-\frac{\sqrt{3}}{2}i_{c} \end{array}\right.$$

After the Clarke transformation has simplified the system into two variables (α-β), these variables are still in the form of alternating current whose values continuously fluctuate in accordance with the motor’s rotation. The Park transformation converts the static reference frame (α-β) into a rotating reference frame (d-q) that is synchronized with the rotor’s rotation. The alternating current is converted into a stable direct current (DC) value. The d-axis (direct) represents the magnetic field (flux), and the q-axis (quadrature) represents torque. The transformation equations are as follows:

(2) $$\left\{ \begin{array}{c} i_{d}=i_{\alpha }cos\theta_{d}+i_{\beta }sin\theta_{d}\\ i_{q}=-i_{\alpha }cos\theta_{d}+i_{\beta }sin\theta_{d} \end{array}\right.$$

After the control system has calculated and determined the voltage to be applied to the d- and q-axes to achieve the desired speed or torque, the Park Inverse Transformation converts the DC control signal from the rotating d-q reference frame back into an AC signal in the two-phase (α-β) stationary reference frame. The transformation equation is shown below:

(3) $$\left\{ \begin{array}{c} V_{\alpha }=V_{d}cos\theta_{d}-V_{q}cos\theta_{d}\\ V_{\beta }=V_{d}cos\theta_{d}-V_{q}cos\theta_{d} \end{array}\right.$$

The inverse Clarke transform then converts the two-phase signal into a three-phase voltage signal (a, b, c), ready to be sent to the inverter to drive the motor. The transformation equation is shown below:

(4) $$\left\{ \begin{array}{c} V_{a}=V_{\alpha }+V_{0}\\ V_{b}=\frac{1}{2}V_{\alpha }+\frac{\sqrt{3}}{2}V_{\beta }+V_{0}\\ V_{c}=\frac{1}{2}V_{\alpha }-\frac{\sqrt{3}}{2}V_{\beta }+V_{0} \end{array}\right.$$

## Mathematical modeling of the BLDC dynamic system

BLDC modeling is often approached using a surface-mounted PMSM mathematical model because the stator current is transformed in a d-q framework. The BLDC model in the d-q framework is shown in the following equation:

(5) $$\left\{ \begin{array}{c} V_{d}=R_{s}i_{d}+L_{d}i_{d}-\omega_{e}L_{q}i_{q}\\ V_{q}=R_{s}i_{q}+L_{q}i_{q}+\omega_{e}L_{d}i_{d}+\omega_{e}\lambda_{f} \end{array}\right.$$

BLDC motor mechanical model:

(6) $$\left\{ \begin{array}{c} J{\dot{\omega }}_{m}=T_{e}-T_{L}-B\omega_{m}\\ J{\dot{\omega }}_{m}=K_{t}i_{q}-T_{L}-B\omega_{m} \end{array}\right.$$

To evaluate the controller gain, the dynamic behavior of a FOC-based Brushless DC (BLDC) motor is mapped into continuous-time differential equations, which are then discretized in a text-based environment. Assuming an ideal trapezoidal back-EMF distribution under FOC, where the d-axis current reference is forced to be zero ($i_{d}^{*}=0$) to maximize torque efficiency, the dynamic electrical equations in the d-q synchronous reference frame are expressed as:

(7) $$\frac{di_{q}\left(t\right)}{dt}=-\frac{R_{s}}{L_{q}}i_{q}\left(t\right)-\frac{\omega_{e}\left(t\right)L_{d}}{L_{q}}i_{d}\left(t\right)+\frac{1}{L_{q}}v_{q}\left(t\right)-\frac{\lambda_{m}}{L_{q}}\omega_{e}\left(t\right)$$

Where $R_{s}$ is the stator resistance, $L_{d}$ and $L_{q}$ are the d-q axis induction, $v_{q}$ and $i_{q}$ are the q axis voltage and current, $\omega_{e}$ is the rotor speed, and $\lambda_{m}$ is the permanent magnet flux linkage. The electromagnetic torque ($T_{e}$) generated in this FOC system is modeled as:

(8) $$T_{e}\left(t\right)=\frac{3}{2}P\lambda_{m}i_{q}\left(t\right)$$

Where P represents the number of pole pairs. The mechanical dynamics governing the rotor acceleration and velocity tracking loop are defined through Newton's second law:

(9) $$\frac{d\omega_{m}\left(t\right)}{dt}=\frac{1}{J}\left(T_{e}\left(t\right)-T_{L}\left(t\right)-B\omega_{m}\left(t\right)\right)$$

Where $\omega_{m}$ is the mechanical rotor speed ($\omega_{e}=P.\omega_{m}$), *J* is the rotor inertia, *B* is the friction coefficient, and $T_{L}$ is the load torque.

In this study, a BLY171D BLDC motor was used, with specifications as shown in Table 1. The motor parameters were obtained from the datasheet.

**Table 1:** BLY171D motor parameters used for the optimization process.

Parameter	Information	Setup value
P	Electrical Poles	4
Rs	Phase Resistance	0.8 Ω
Ld, Lq	Phase Inductance	1.2 mH
J	Inertia	4.8 x $10^{-8}Kg-m^{2}$
B	Friction coefficient	$1\;x\;10^{-4}Kg-m^{2}/s$
Ke	Back EMF const	$32\;x\;10^{-3}$ Nm/A
Kt	Torque constant	$32\;x\;10^{-3}$ Nm/A
ω	Rated speed	4000 RPM

## Modeling of conventional PI speed controller

Next, an important aspect that needs to be explained to provide a more comprehensive picture of the interactions between parameters and signal flow in a control system is the implementation of a PI controller, whose output is generally expressed as:

(10) $$u\left(t\right)=K_{p}e\left(t\right)+K_{i}\int e\left(t\right)dt$$

Where $K_{p}$ is the proportional gain, $K_{i}$ is the integral gain, and *e(t)* is the error signal between the reference value and the actual value of the system.

**Fig. 3:**
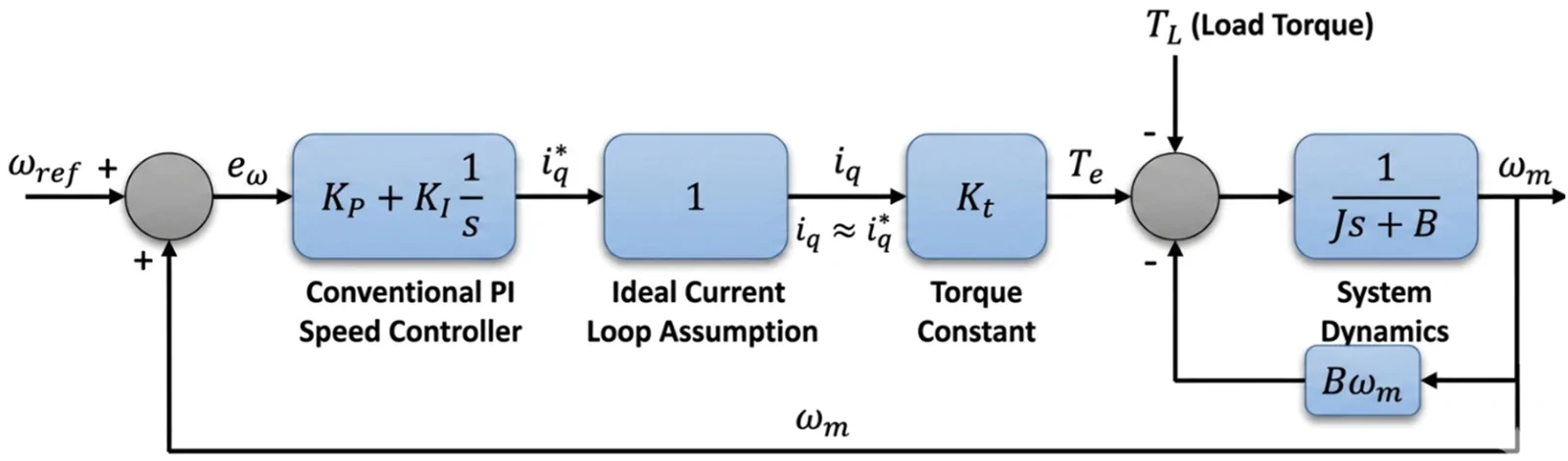
Block diagram of PI speed controller.

This control method is commonly used in motor speed control systems because it has a simple structure and can reduce steady-state error. The following flowchart of the speed control block is shown in Fig. 3.

The speed error is defined as the difference between the reference speed ($\omega_{ref}$) and the actual motor speed ($\omega_{m}$) fed back from the sensor:

(11) $$e_{\omega }=\omega_{ref}-\omega_{m}$$

To eliminate the steady-state error, the error signal is then integrated to obtain the integral error variable ($e_{\omega }$) which then becomes the input to the PI controller. In this block, the control action equation is represented by the transfer function in the s-domain (Laplace):

(12) $$\left\{ \begin{array}{c} z=\int e_{\omega }dt\\ K_{p}+K_{i}\frac{1}{s} \end{array}\right.$$

The output of this PI block is the quadrature-axis current command signal ($i_{q}^{*}$), which represents how much current is required to achieve the target speed and can be written as:

(13) $$i_{q}^{*}=K_{p}e_{\omega }+K_{i}z$$

The resulting $i_{q}$ value is then converted into a mechanical quantity in the form of electromagnetic torque ($T_{e}$). This relationship is bridged by the motor torque constant ($K_{t})$, where its value is linear with the current at optimal FOC conditions ($i_{d}=0$). The equation is:

(14) $$T_{e}=K_{t}.i_{q}$$

Before generating motion, the electromagnetic torque ($T_{e})$ must counteract external and internal disturbances on the motor shaft. At the second summation point, a physical reduction occurs:

• $T_{L}$ (Load Torque): External mechanical load applied to the motor (e.g. vehicle or engine load).
• $B\omega_{m}$ (Viscous Friction): The resistance torque due to internal mechanical friction and lubricating bearings whose value is proportional to the current motor speed.

So the mechanical dynamics of the motor can be expressed as:

(15) $$J{\dot{\omega }}_{m}=K_{t}\left(K_{p}e_{\omega }+K_{i}z\right)-T_{L}-B\omega_{m}$$

Where J is the rotor moment of inertia, $K_{t}$ the motor torque constant, $T_{L}$ the load torque, and B the viscous friction coefficient.

## Initialization and search dimension configuration

Before executing an iterative search cycle, the physical search boundaries and operational environment of the drive are established through a setup routine:

### Definition of parameter limits

To enforce realistic search space constraints on the controller output and avoid unstable tracking modes, the search dimension coordinates are setup:

(16) $$\left\{ \begin{array}{c} K_{p,min}\leq K_{p}\leq K_{p,max}\\ K_{i,min}\leq K_{i}\leq K_{p,max} \end{array}\right.$$

### Operational conditions and per-unit system

The per-unit (PU) system is used in MATLAB simulations to normalize electrical and mechanical variables, thereby reducing numerical stiffness and simplifying controller tuning. Typically, the system uses its nominal value as the base value. In some cases, the system may also set the maximum measured value as the base value. The general convention for determining the base value in pu units is shown in the following equation:

(17) $$quantity\;expressed\;in\;PU=\;\frac{quantity\;expressed\;in\;SI\;units}{base\;value}$$

Because the rated speed of the motor used is 4000 RPM, the pu unit conversion is:

(18) $$Speed\;\left(PU\right)=\frac{4000\;RPM\;\left(SI\right)}{4000\;RPM\;\left(Base\right)}=1░\text{pu}$$

To tune and evaluate the controller specifically for high-speed dynamic performance, a constant reference speed profile ($\omega_{ref}$) is applied uniformly across all evaluations to be:

(19) $$\omega_{ref}=0.8\;PU$$

## Step-by-Step computational execution sequence

The detailed operational workflow of the auto tuning process runs sequentially and chronologically as described below:

### Step 1: population initialization

The Core Optimization Module defines the total population size (N gray wolves) and the maximum allowed iterations (T). The position vector Xi = [Kp,i, Ki,i] for each search agent is randomly generated within the safe operational search space boundary.

**Figure:**
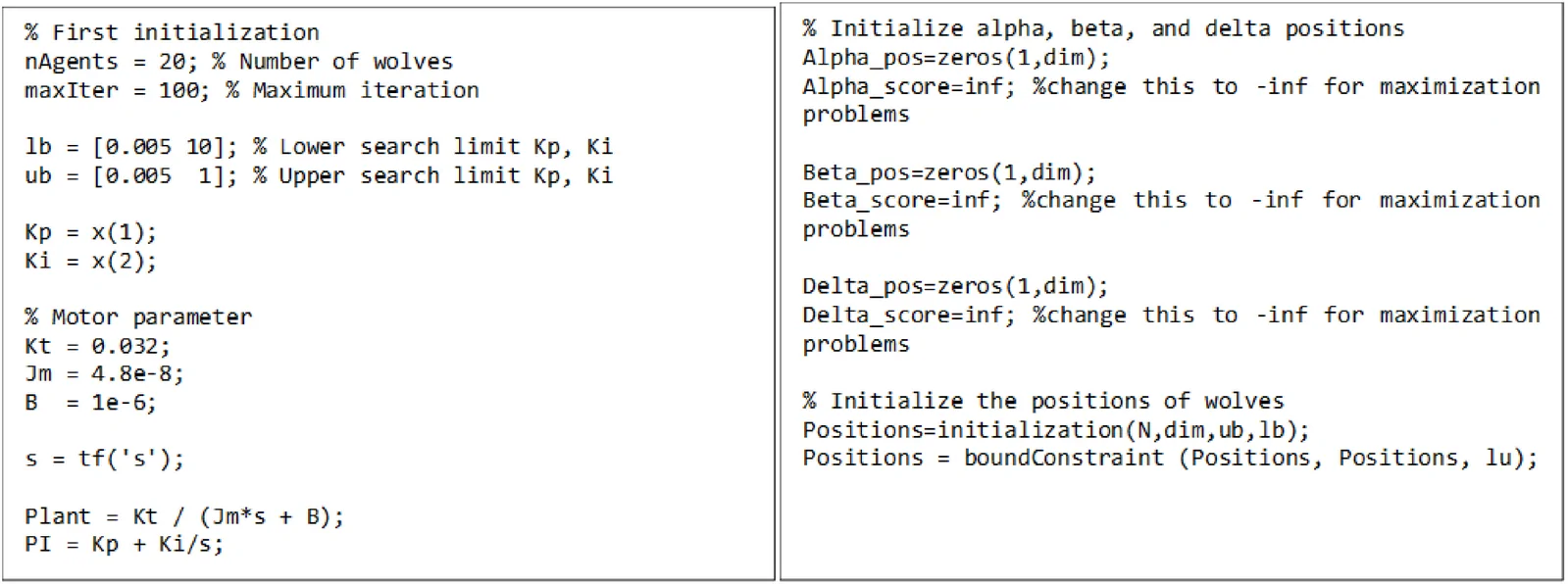

### Step 2: mathematical transient and fitness evaluation

For each individual agent *i* in the current population, its coordinate parameters ($K_{p,i}$ dan $K_{i,i}$) are fed into the Objective Function Evaluation Module. This module integrates these gains into the discretized transfer function of the system or the state-space matrix. A numerical solver loop then simulates the dynamic step response under a constant high-speed reference profile of 0.8 pu for a specified time-domain duration ($t_{sim}$). The resulting operational tracking quality is evaluated by computing a multi-objective cost function that combines a modified Integral of Time-weighted Absolute Error (ITAE) with a strict penalty for transient overshoot ($M_{p}$) and control energy consumption:

(20) $$fitness=\int_{0}^{t_{sim}}\left(t.\left|e\left(t\right)\right|\right)dt+\omega_{1}.M_{p}+\omega_{2}.\int_{0}^{t_{sim}}{\left[u\left(t\right)\right]}^{2}dt$$

Where:

• $e\left(t\right)=\omega_{ref}-\omega_{act}\left(t\right)$ represents the instantaneous speed reading error in Per-Unit (PU).
• u(t) is the control effort generated by the PI speed loop (serves as an internal torque or current command reference).
• $\omega_{1}$ and $\omega_{2}$ represent scalar weighting factors adjusted to balance tracking precision with real-world physical stress and system overshoot.

**Figure:**
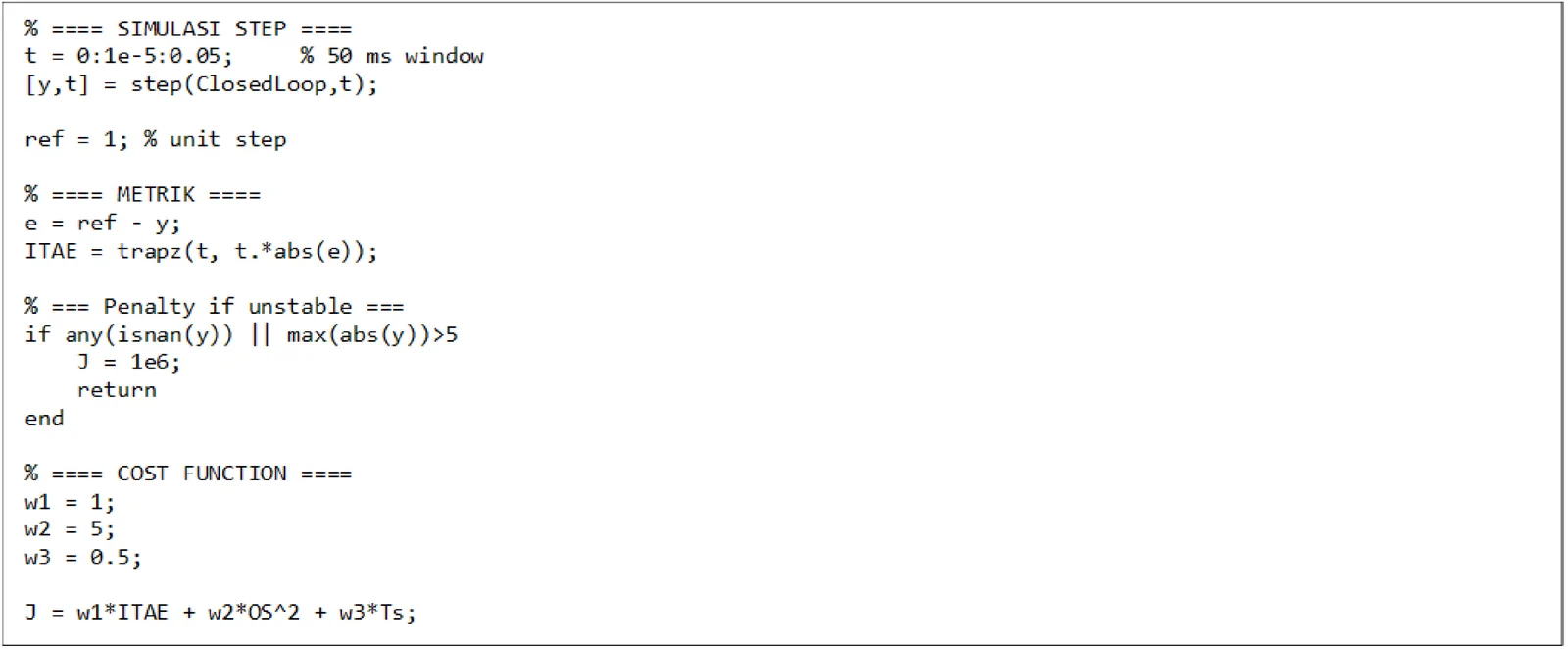

### Step 3: hierarchical sorting and leader assignment

The fitness scores calculated for the entire swarm are fed back to the Core Optimization Module. The population is sorted in ascending order to identify three dominant candidate solutions:

$X_{\alpha }$: The position vector that produces the lowest overall cost function value (agent Alpha).
$X_{\beta }$: The second best performing position vector (agent beta).
$X_{\delta }$: The third best performing position vector (Delta agent).

**Figure:**
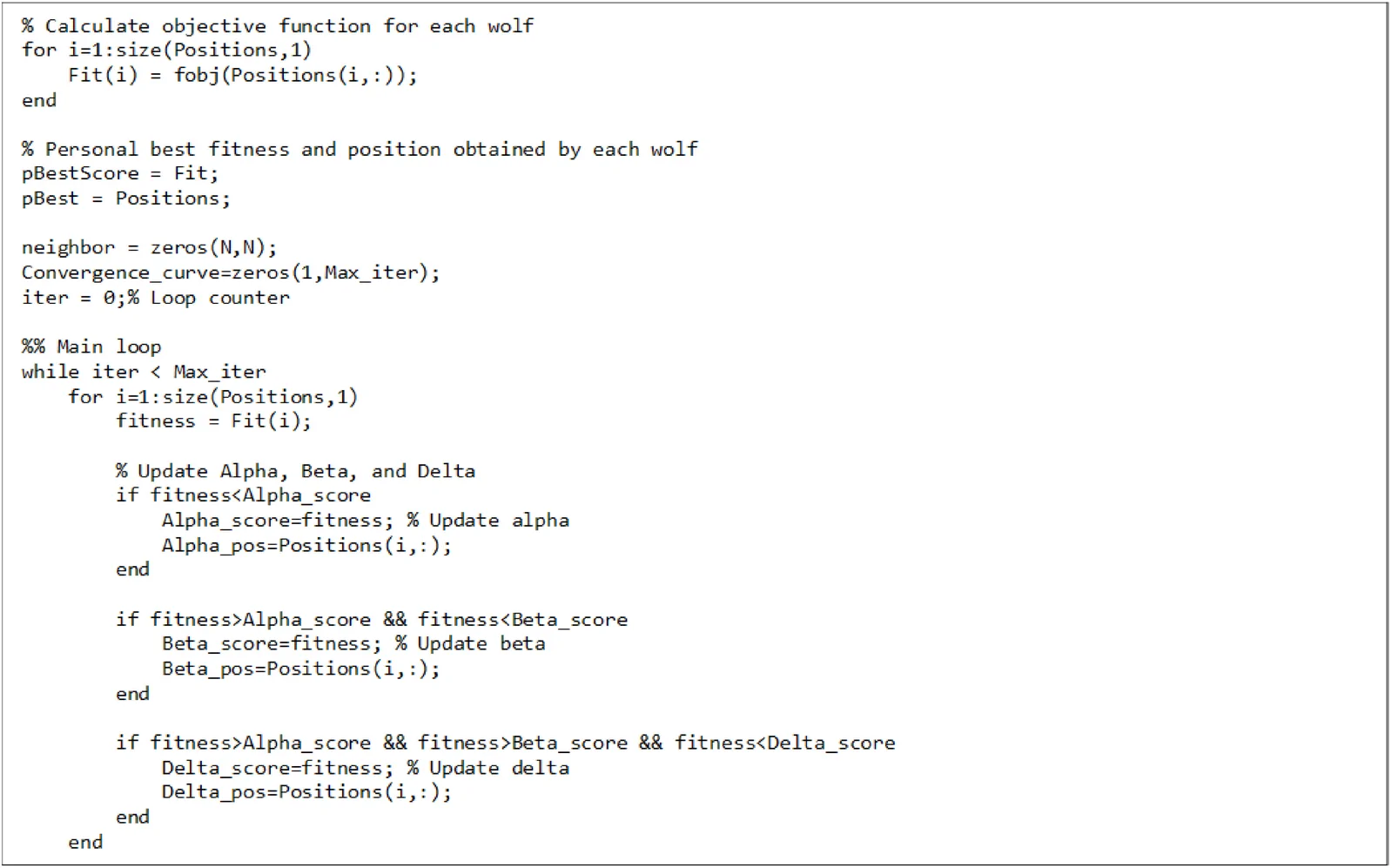

### Step 4: vector position updating mechanism

In the hunting behavior carried out by gray wolves, they will first carry out an encirclement, for which the mathematical modeling is shown in the following equation:

(21) $$\vec{D}=\left|\vec{C}.\vec{X_{P}}\left(t\right)-\vec{X}\left(t\right)\right|$$

(22) $$\vec{X}\left(t+1\right)=\vec{X_{P}}\left(t\right)-\vec{A}.\vec{D}$$

Where t denotes the current iteration, $\vec{A}$ and $\vec{C}$ are coefficient vectors, ($\vec{X_{p}}$) is the prey position vector, and $\vec{X}$ denotes the gray wolf position vector. The vectors $\vec{A}$ and $\vec{C}$ are calculated using the equations below:

(23) $$\vec{A}=2\vec{a}.\vec{r_{1}}.\vec{a}$$

(24) $$\vec{C}=2\vec{r_{2}}$$

The spatial coordinates of all search agents in the two-dimensional search grid (Kp x Ki) are updated. This hunting simulation mimics cooperative tracking by modifying the agents' paths relative to the leader:

(25) $$D_{\alpha }=\left|C_{1}.X_{\alpha }-X\right|,D_{\beta }=\left|C_{2}.X_{\beta }-X\right|,D_{\delta }=\left|C_{3}.X_{\delta }-X\right|$$

(26) $$X_{1}=X_{\alpha }-A_{1}.D_{\alpha },X_{2}=X_{\beta }-A_{2}.D_{\beta },X_{3}=X_{\delta }-A_{3}.D_{\delta }$$

The resulting position update for a given agent at the next sequence (t + 1) is determined as the geometric mean of the primary target trajectories:

(27) $$X\left(t+1\right)=\frac{X_{1}+X_{2}+X_{3}}{3}$$

The coefficient vectors A and C are reduced linearly during the iterations to control the balance between global exploration and local exploitation.

**Figure:**
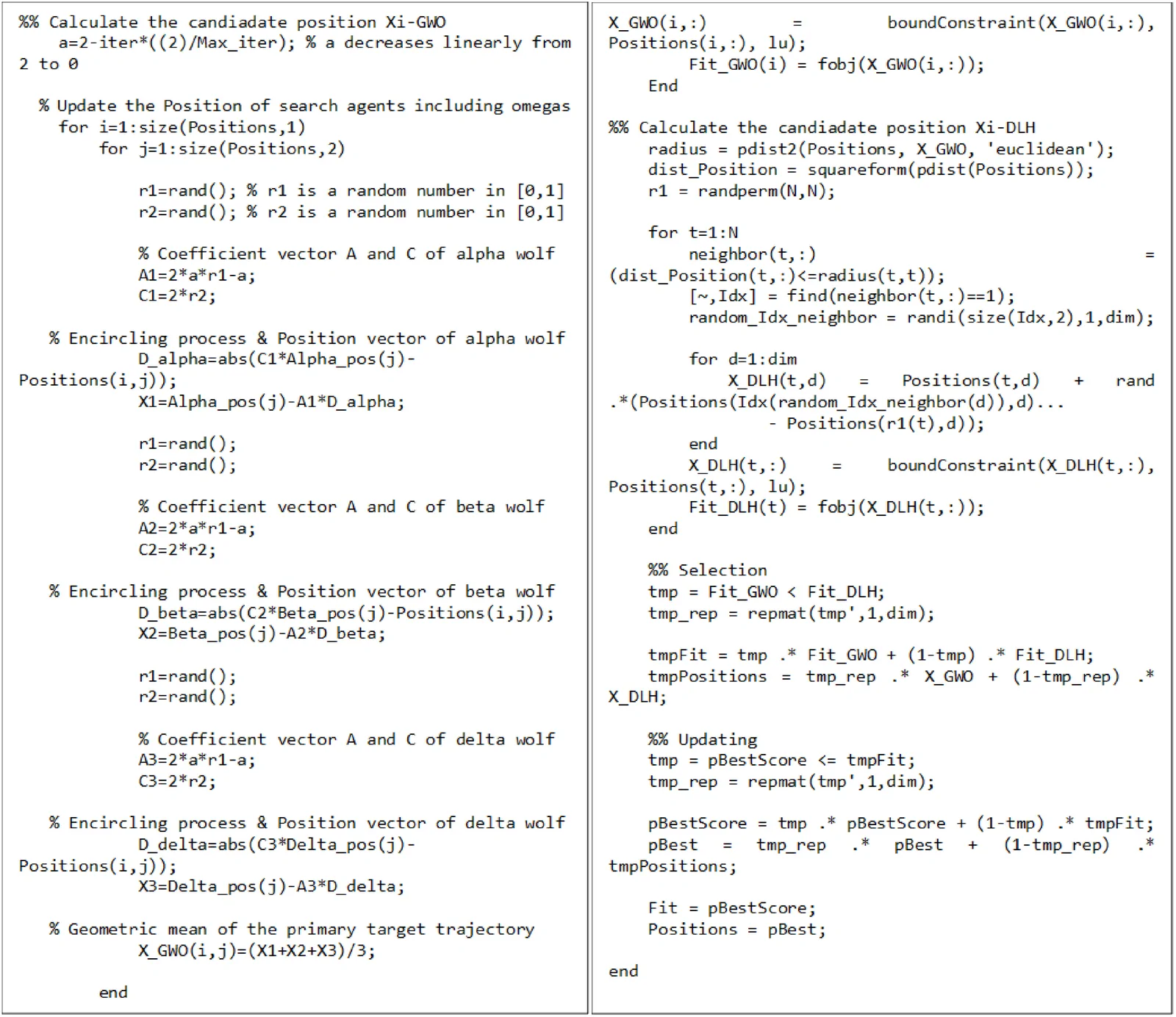

### Step 5: boundary constraint mechanism

During the optimization process, candidate solutions generated by GWO may sometimes violate the predefined search bounds of decision variables. This situation is common in population-based metaheuristic algorithms due to the stochastic exploration mechanisms used to update agent positions. Therefore, a mechanism for handling boundary constraints is necessary to ensure that the generated solutions remain within a feasible search space.

Let $X_{i}\in R^{D}$ denote the position vector of the i th search agent in the d-dimensional decision space. Each dimension is bounded by lower and upper limits defined as:

(28) $$I=\left[l_{1},l_{2},\ldots .,l_{D}\right],\;u=\left[u_{1},u_{2},\ldots .,u_{D}\right]$$

Where $l_{d}$ and $u_{d}$ represent the lower and upper bounds of the d-th variable, respectively.

When an updated candidate solution $v_{i}$ violates this constraint, a corrective strategy is applied. Instead of immediately clipping the value to the violated bound, the proposed approach moves the candidate solution to the midpoint between the previous feasible position and the violated bound. This strategy helps maintain population diversity and avoids premature convergence that might occur when many agents cluster right at the boundary. For a violated lower bound, the correction is defined as:

(29) $$v_{i,d}=\frac{x_{i,d}+l_{d}}{2},\;ifv_{i,d}<l_{d}$$

Similarly, if the candidate solution exceeds the upper bound, the correction becomes:

(30) $$v_{i,d}=\frac{x_{i,d}+u_{d}}{2},\;ifv_{i,d}<u_{d}$$

**Figure:**
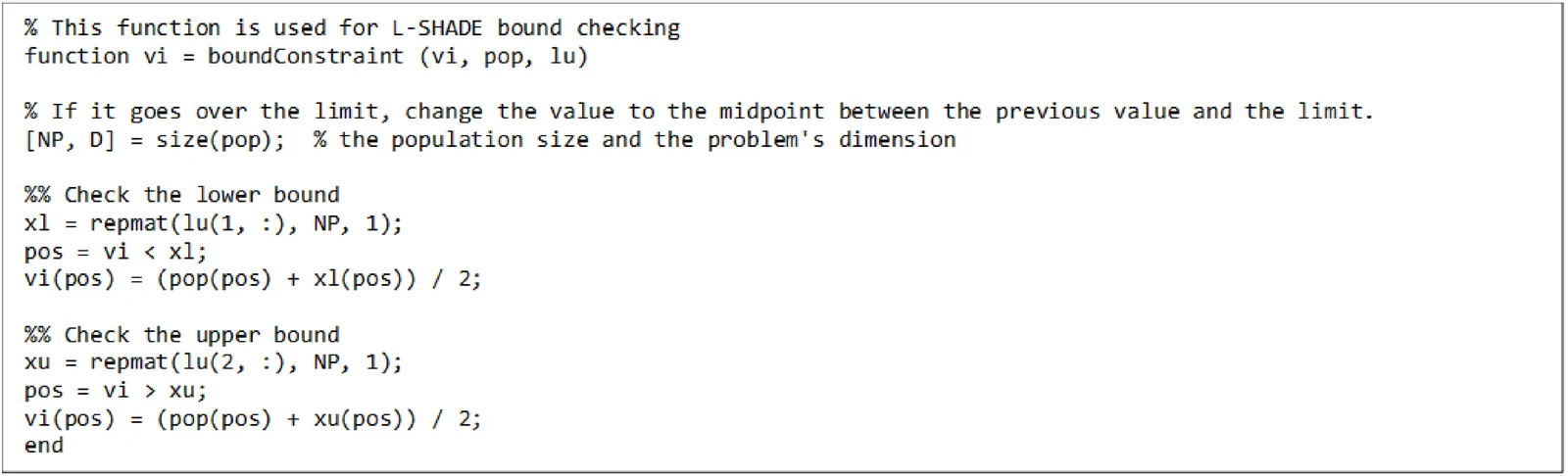

### Step 6: iterative termination and parameter export

The global iteration counter is incremented (t = t + 1). Steps 2 through 5 are performed iteratively. The cycle ends exactly when t = T. are exported as the absolutely adjusted control gains $(K_{p}^{*}$ and $K_{i}^{*})$ for final implementation in the FOC drive system.

**Figure:**
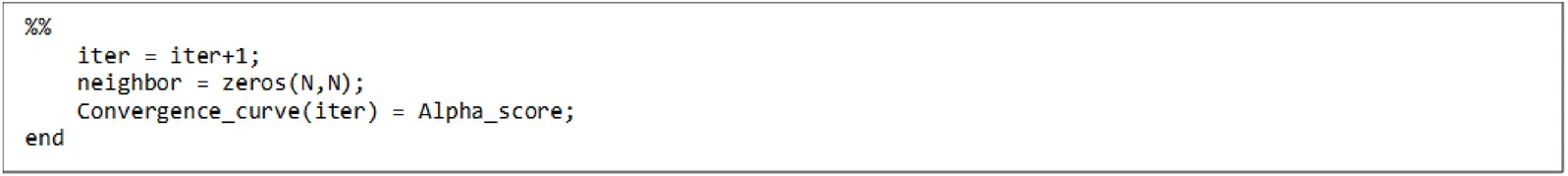

## Method validation

In this section, the performance and effectiveness of the method described in the previous chapter for optimizing the PI controller in the field-oriented control of a BLDC using GWO are demonstrated. The performance of the control system is evaluated based on time-domain speed response characteristics, including rise time, overshoot, settling time, and steady-state error. The analysis begins with an examination of the convergence behavior of the GWO algorithm, followed by a comparative evaluation of the FOC speed response with and without GWO optimization under no-load and loaded conditions.

In addition, to validate the results in practice, an experiment was conducted using a TMS320F28035 Digital Signal Processor (DSP) board and a BLY171D BLDC motor with the specifications described in Table 1. The entire process was designed and programmed using MATLAB/Simulink.

## Convergence behavior of GWO algorithm

**Fig. 4:**
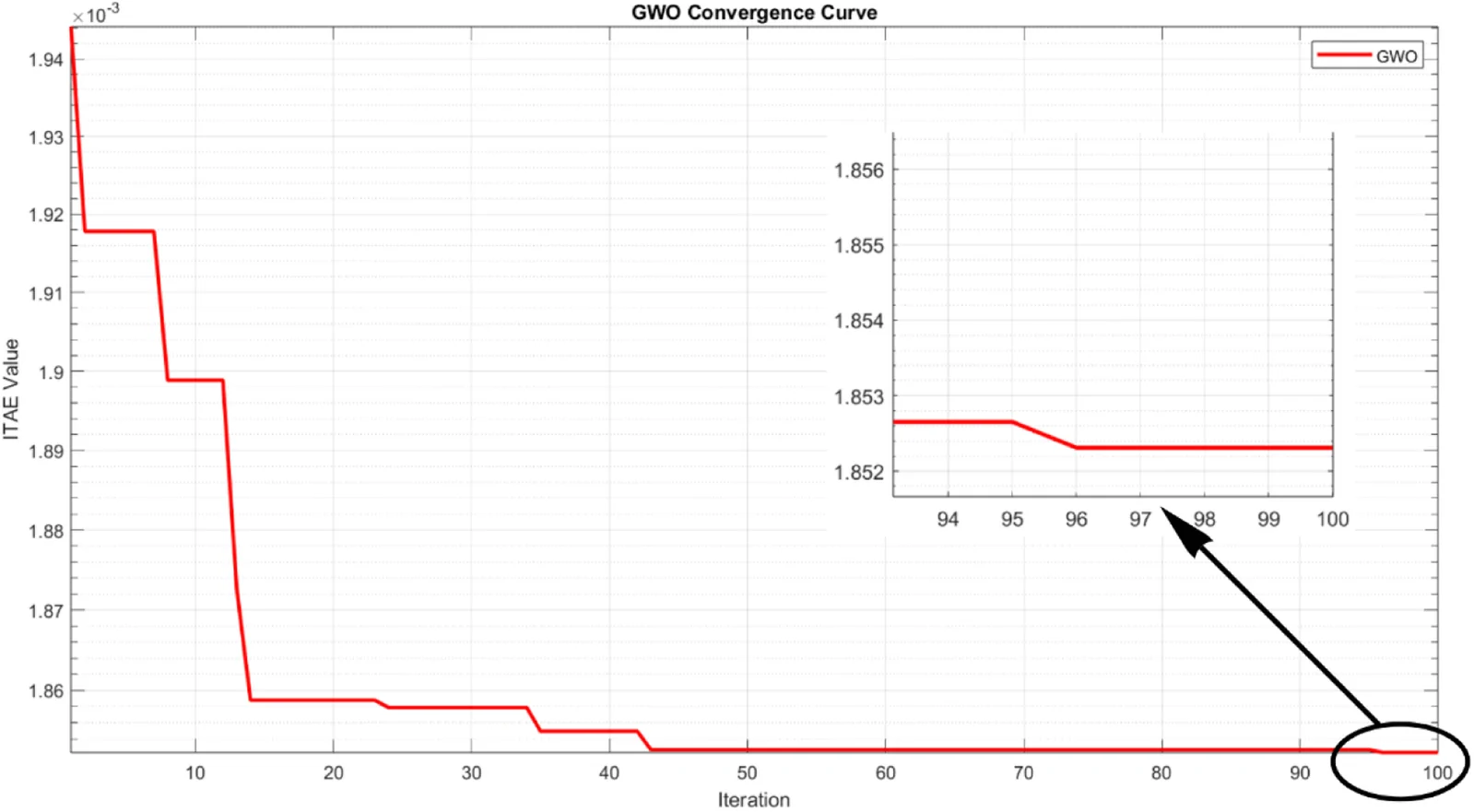
GWO Convergence results.

The results of the GWO experiment using MATLAB produced a convergence plot as shown in Fig. 4. The convergence curve shows a gradual decrease in the objective function value as the number of iterations increases, indicating that the GWO algorithm successfully searches toward a more optimal solution. In the early iterations, a significant and rapid decrease in the fitness value is observed. This indicates that the algorithm is extensively exploring the solution space. After the initial exploration phase, the curve begins to show a slight and more gradual decrease. This indicates that the algorithm is shifting from exploration to exploitation of the solutions it has found. After around the 45th iteration, the curve begins to flatten, and changes in the fitness value become very small. This indicates that the algorithm has entered an intensive exploitation phase around the best solution it has found. The fitness value begins to stabilize around $1.852\;\times 10^{-3}$. Zooming in on iterations 94–100 shows that changes in the objective value are very small and nearly constant. This indicates that the algorithm has reached a converged state.

## Computational cost and parameter sensitivity

The computational cost of the proposed GWO-based tuning procedure is primarily determined by the population size (N) and the maximum number of iterations (T), since each iteration requires N evaluations of the fitness function in Eq. (20), and each evaluation involves the numerical solution of the closed-loop system’s transient response. Therefore, the overall computational complexity can be estimated using Big-O notation to express how quickly an algorithm’s computational cost grows as its parameters increase, and is expressed as O(N × T × Cf), where Cf represents the cost of a single transient simulation and fitness calculation. In this study, the population size was set to N = 20 search agents and the maximum number of iterations to T = 100. These values were chosen because the decision space involves only two variables (Kp and Ki), and a medium population size is generally sufficient to effectively cover a low-dimensional search space.

As shown by the convergence curve in Fig. 4. the fitness value decreases sharply during the first 45 iterations or so before leveling off, and the change after the 94th iteration is very small. This indicates that the algorithm effectively converges well before the maximum number of iterations is reached, while also providing empirical evidence that the chosen value of T = 100 provides a sufficient margin for convergence in this application, and that further increasing the value of T would only add to the computation time without a commensurate improvement in solution quality.

To empirically assess the sensitivity of the algorithm to these parameters, an ablation study was conducted by varying the population size and the maximum number of iterations within the ranges N ∈ {10, 15, 30} and T ∈ {50, 75, 150}, resulting in nine combinations. Each combination was tested through 10 independent runs. Since GWO is a stochastic algorithm, the final fitness values and computation times obtained were averaged across these 10 runs for each combination. The results are summarized in Table 2.

**Table 2:** Comparison results: mean final fitness and computation time across N and T combinations (10 runs each).

Total population (N)	Iteration (T)	Mean fitness	Std fitness	Mean time (s)
10	50	0.0018,586	9.70×10⁻⁶	2.92
10	75	0.0018,557	8.07×10⁻⁶	4.25
10	150	0.0018,548	4.21×10⁻⁶	8.45
15	50	0.0018,548	2.12×10⁻⁶	4.22
15	75	0.0018,524	1.57×10⁻⁶	6.19
15	150	0.0018,526	1.66×10⁻⁶	12.28
30	50	0.0018,542	2.91×10⁻⁶	8.24
30	75	0.0018,520	2.41×10⁻⁶	12.28
30	150	0.0018,506	6.99×10⁻⁶	24.55

From nine combinations tested, the average final fitness showed only very small variations, ranging from 0.0018,506 to 0.0018,586; this indicates that the quality of solutions from the proposed framework is essentially insensitive to the choice of N and T values within the tested range. Std fitness (standard deviation of fitness values) represents the standard deviation of the final fitness values obtained from 10 independent runs for each (N, T) combination; this value reflects the inter-run variability resulting from the random initialization in the GWO algorithm. It is observed that the Std fitness value decreases as N increases, ranging from a maximum value of 9.70×10⁻⁶ at N = 10, T = 50 to a minimum value of 6.99×10⁻⁷ at N = 30, T = 150, a decrease of nearly 14-fold. This trend indicates that smaller populations are more sensitive to the randomness of the wolf pack’s initial positions, sometimes causing them to converge to slightly different local solutions. Conversely, larger populations explore the search space more thoroughly and converge more consistently across different runs.

**Fig. 5:**
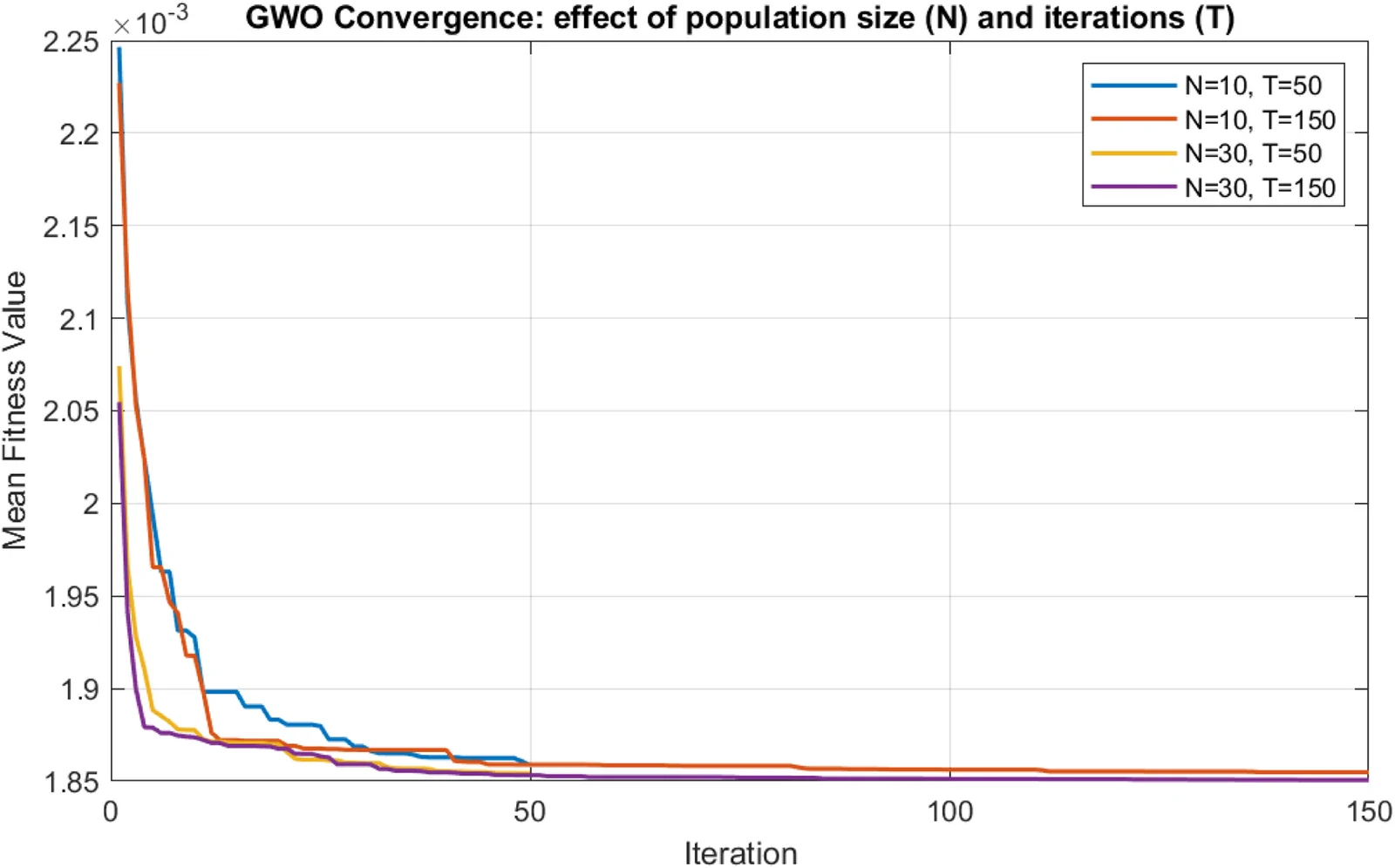
GWO convergence curves for varying population size (N) and maximum iteration count (T).

Fig. 5. illustrates the averaged convergence behavior from 10 trials for four representative combinations at the extreme points of the tested range (N = 10, T = 50; N = 10, T = 150; N = 30, T = 50; N = 30, T = 150). During the first 40–50 iterations, the curves appear separated, reflecting differences in exploration behavior due to differences in population size.

**Fig. 6:**
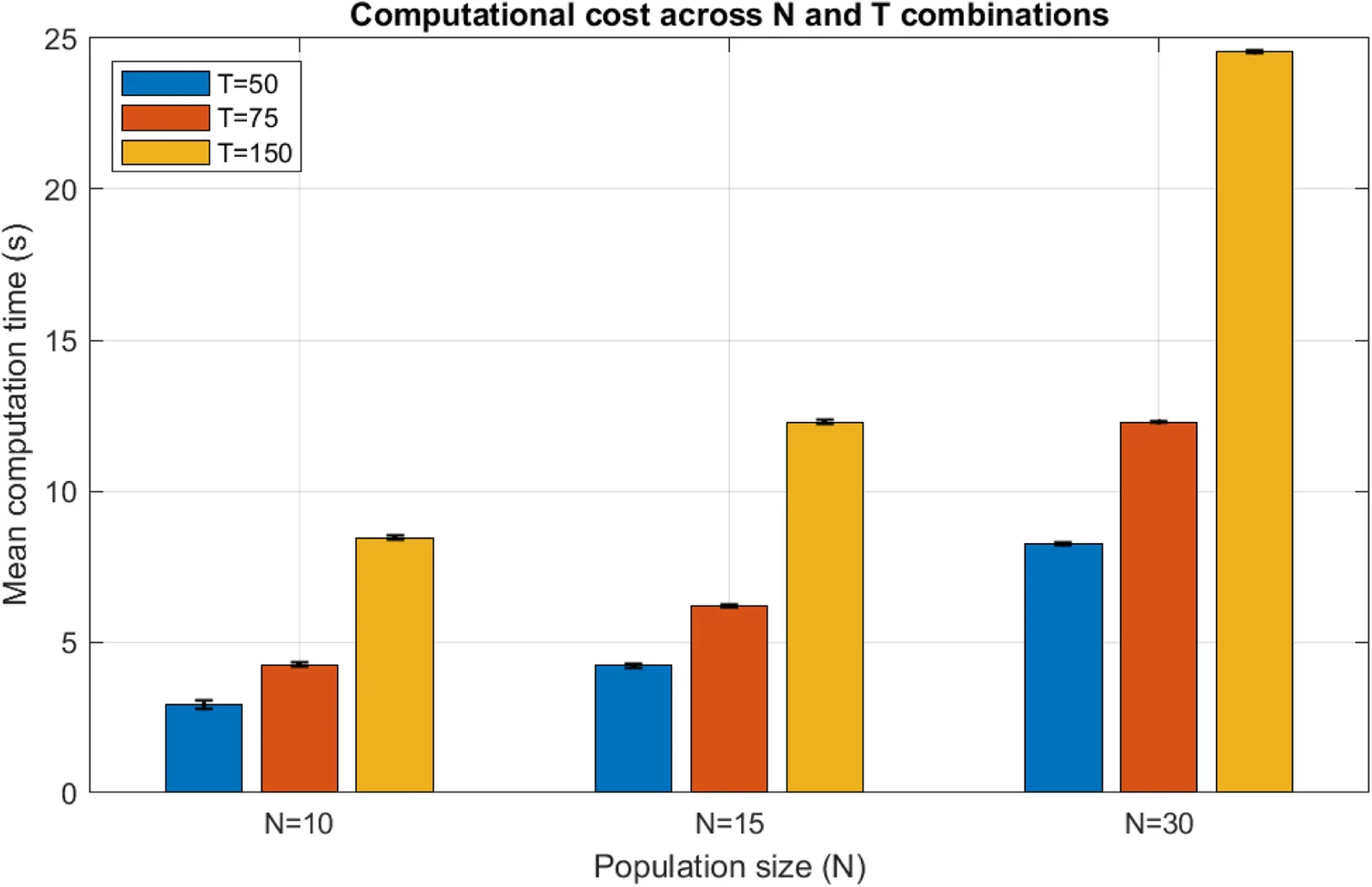
Mean computation time per (N, T) combination.

Fig. 6. shows that the average computation time increases proportionally with N × T, ranging from approximately 2.9 s (N = 10, T = 50) to 24.5 s (N = 30, T = 150) per run. These results indicate that, for this application, increasing the population size or the number of iterations beyond moderate values yields only a negligible improvement in solution quality, yet causes a proportional increase in computational cost, this supports the use of moderate population sizes and iteration budgets for the offline tuning phase. This lack of sensitivity should not be interpreted as a general property of metaheuristic optimization, rather, it reflects the characteristics of a low-dimensional (two-variable) problem that is not overly complex. For problems with higher dimensions or those that are more challenging, population size and the number of iterations are expected to have a much stronger influence on solution quality. therefore, the moderate settings validated here cannot be assumed to be directly applicable to such cases.

## Analysis of the FOC response without GWO optimization

**Fig. 7:**
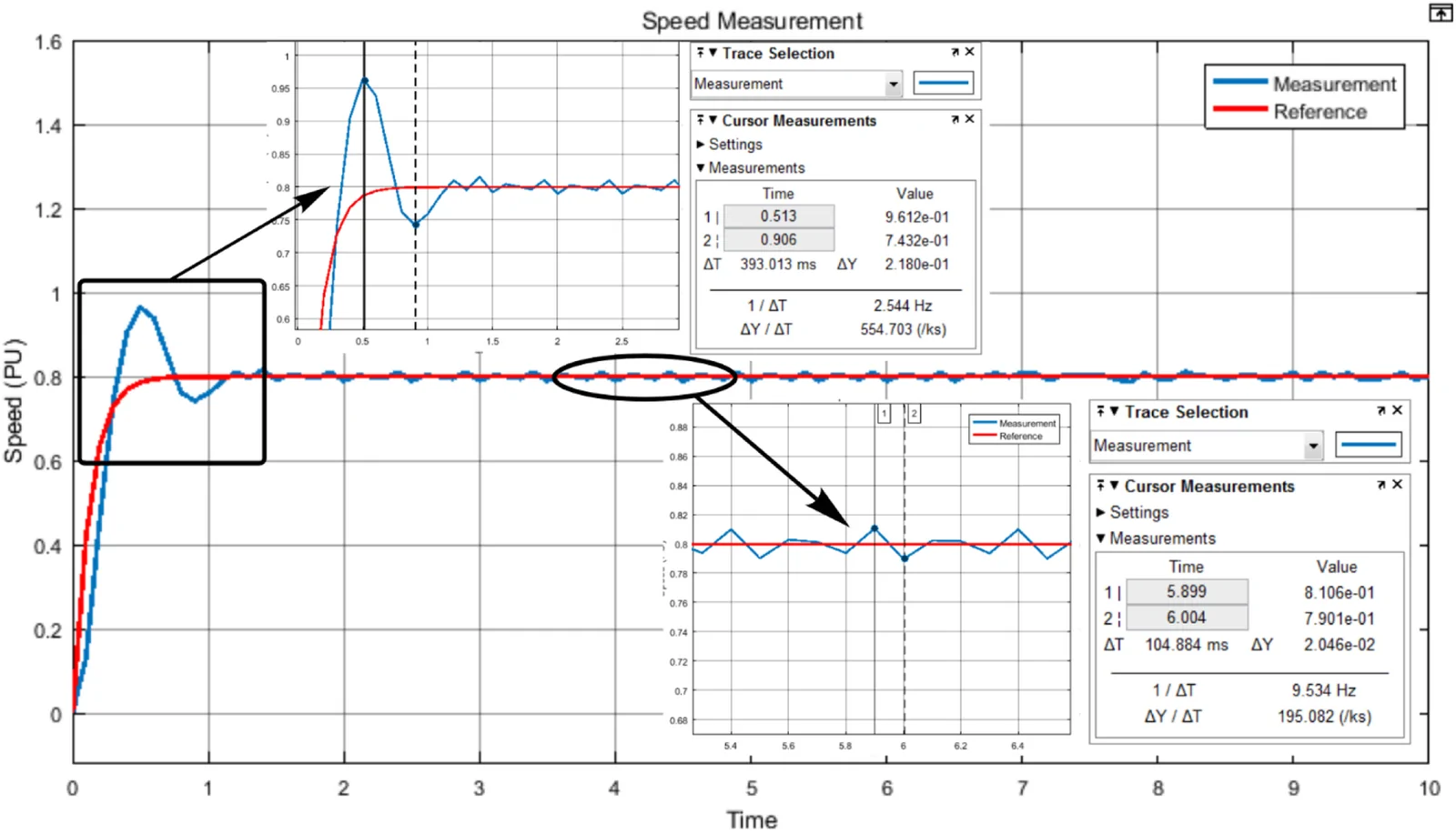
FOC speed response without load condition.

**Fig. 8:**
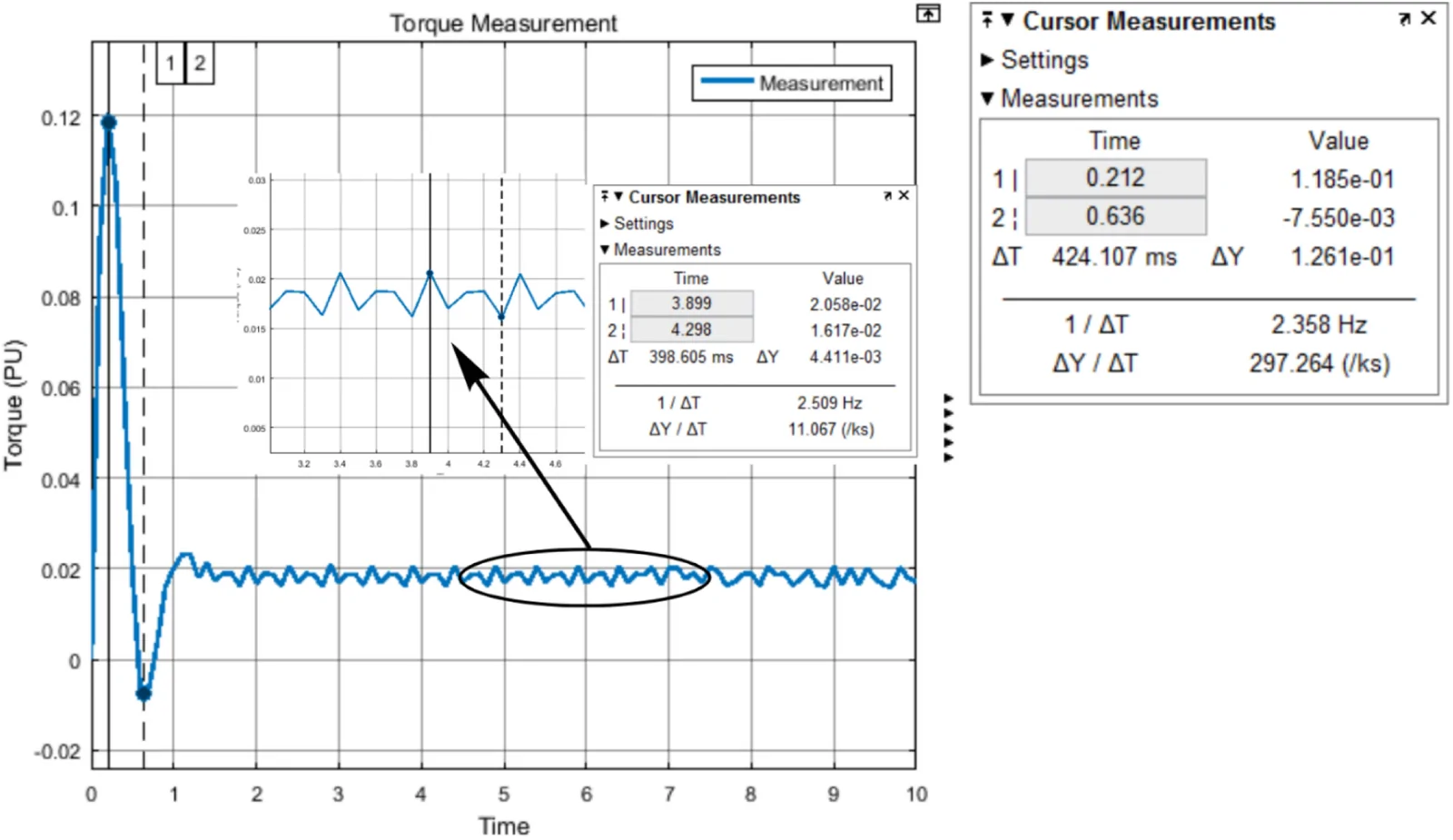
FOC torque response without load condition.

**Fig. 9:**
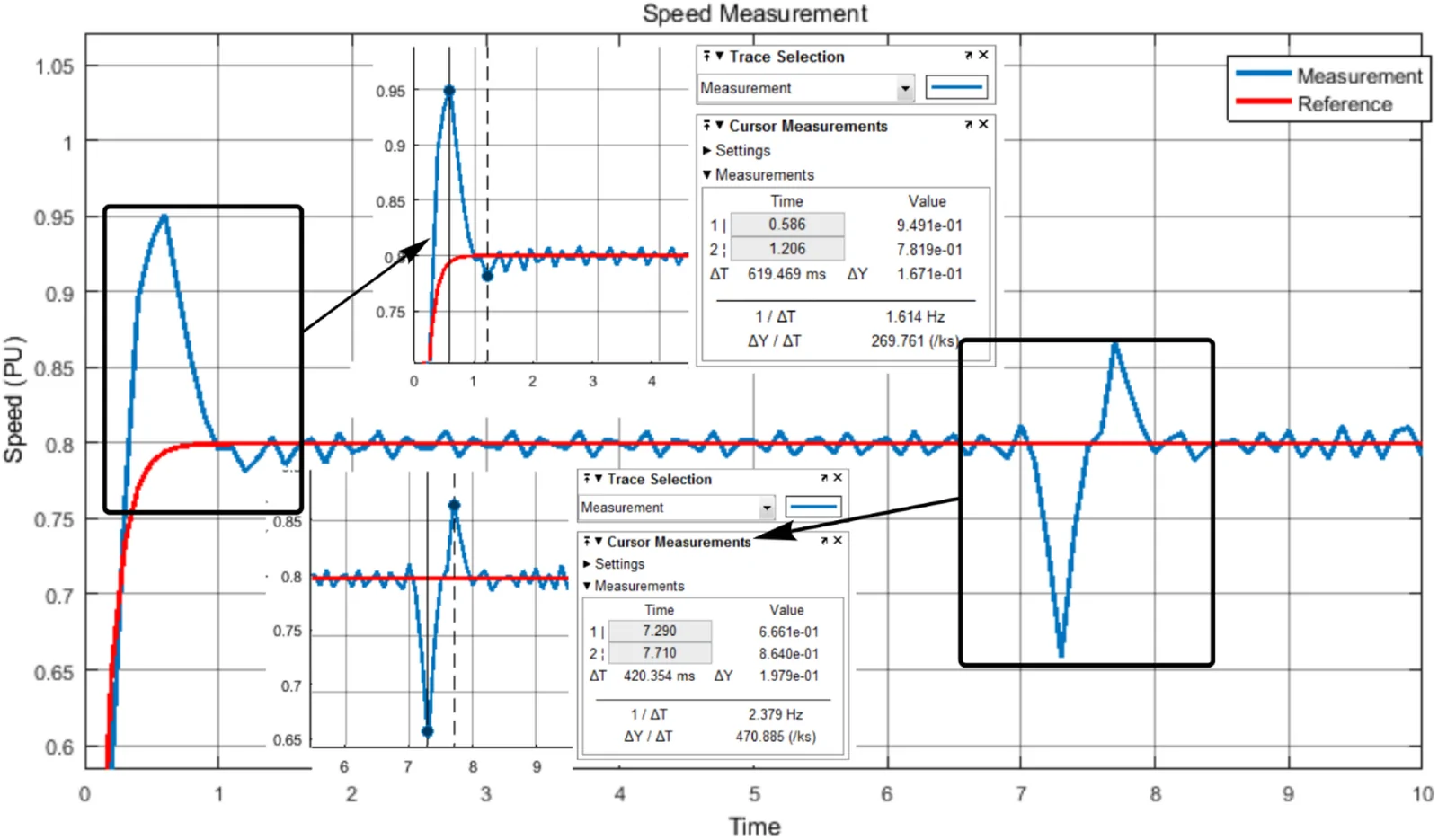
FOC speed response under load conditions.

**Fig. 10:**
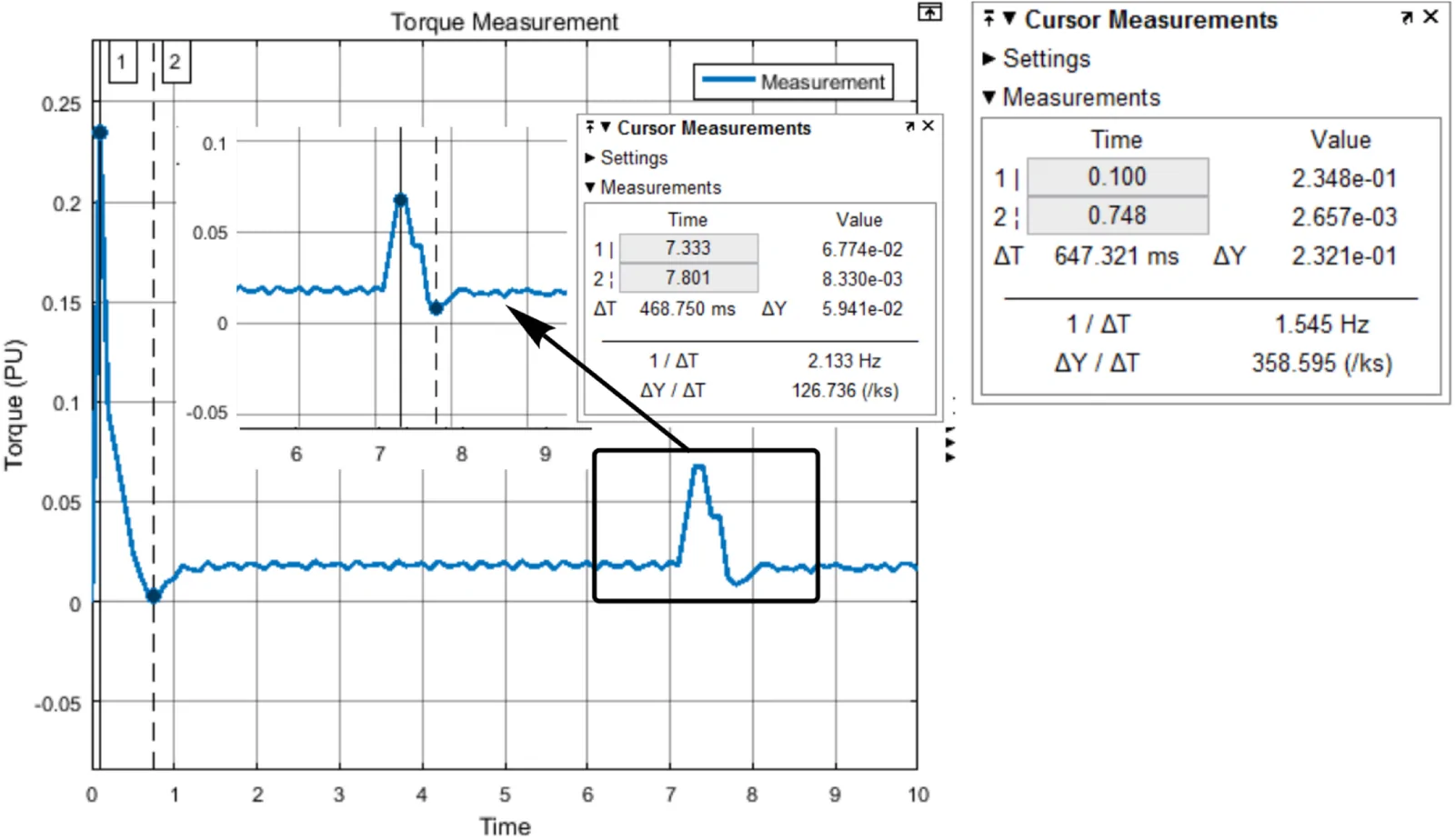
FOC torque response under load condition.

This section presents an analysis of the speed and torque responses of the FOC motor without GWO-optimized PI parameters, both under no-load conditions and under load disturbances, as shown in Fig. 7, Fig. 8, Fig. 9, Fig. 10.

### Response under no-load conditions

The speed response of the motor using conventional FOC is shown in Fig. 7. The system exhibits a fast rise time (±0.35 s) and reaches steady state in approximately 1.2 s. Although there is an overshoot of 20.15%, the system still reaches steady state with very small steady-state error (<0.05%). In the steady-state condition, there is a small ripple of approximately 0.02 pu, which is still within acceptable limits for an inverter-based motor drive system. Regarding the resulting torque response, Fig. 8. shows that the conventional FOC produces a large initial torque surge (±0.1185 pu) during the startup phase to accelerate the rotor toward the reference speed. After reaching the maximum value, the system experiences a small torque undershoot (±−0.00,755 pu) before finally entering a stable condition. The transient phase lasts approximately 0.424 s, after which the system reaches a steady-state condition with an average torque value of about 0.018 pu. In this condition, a small torque ripple is observed, caused by the inverter switching characteristics and the digital control process.

### Response under load disturbance conditions

Fig. 9. shows the speed response of a conventional FOC system under load disturbance conditions, demonstrating a rapid speed response during startup, with a rise time of approximately 0.30 s and a settling time of approximately 1.1 s. The system also exhibits an overshoot of 18.75%, indicating underdamped response characteristics. When the load is applied at approximately 7.3 s, the motor speed drops by 0.66 pu, or about 17.5% of the reference value. This indicates that conventional FOC control still has limitations in maintaining speed during sudden load changes. Nevertheless, the system recovers the speed to the reference value in about 0.6 s, with a recovery overshoot of 7.5%.

The torque response graph is shown in Fig. 10. The test results indicate that the conventional FOC control can produce a sufficiently fast torque response in response to changes in operating conditions. During the startup phase, the system generates a significant initial torque surge (±0.24 pu) to accelerate the rotor to the reference speed. After the transient phase ends, the system enters a steady-state condition with a relatively small torque value (±0.02 pu) and low ripple. When a sudden load disturbance was applied at approximately 7.1 s, the system increased torque by ±0.07 pu to compensate for the additional load on the motor. Despite this significant increase in torque, the system returned to a stable state within approximately 0.6 s.

## Analysis of the FOC response using GWO optimization

**Fig. 11:**
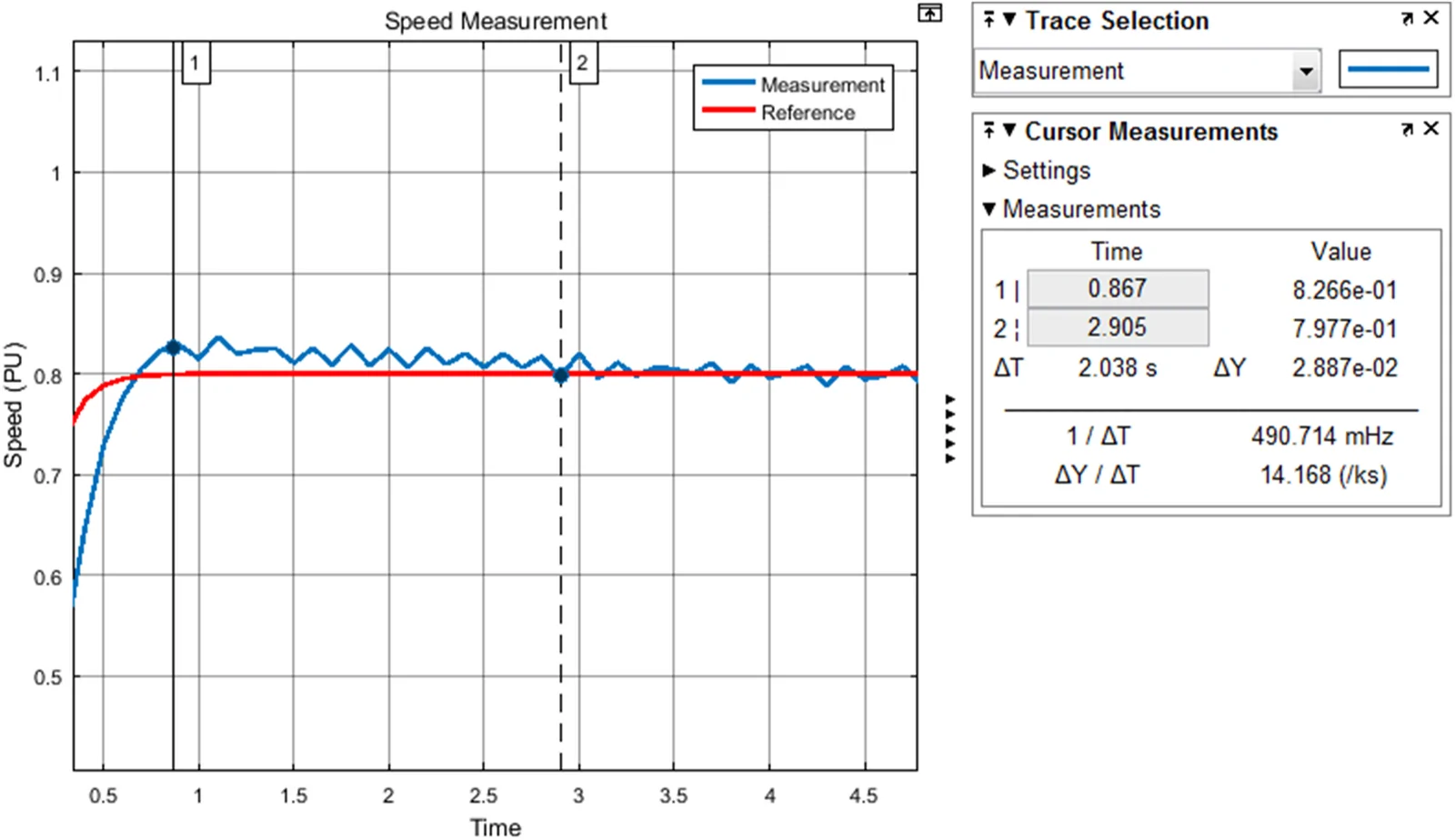
FOC-GWO speed response without load condition.

**Fig. 12:**
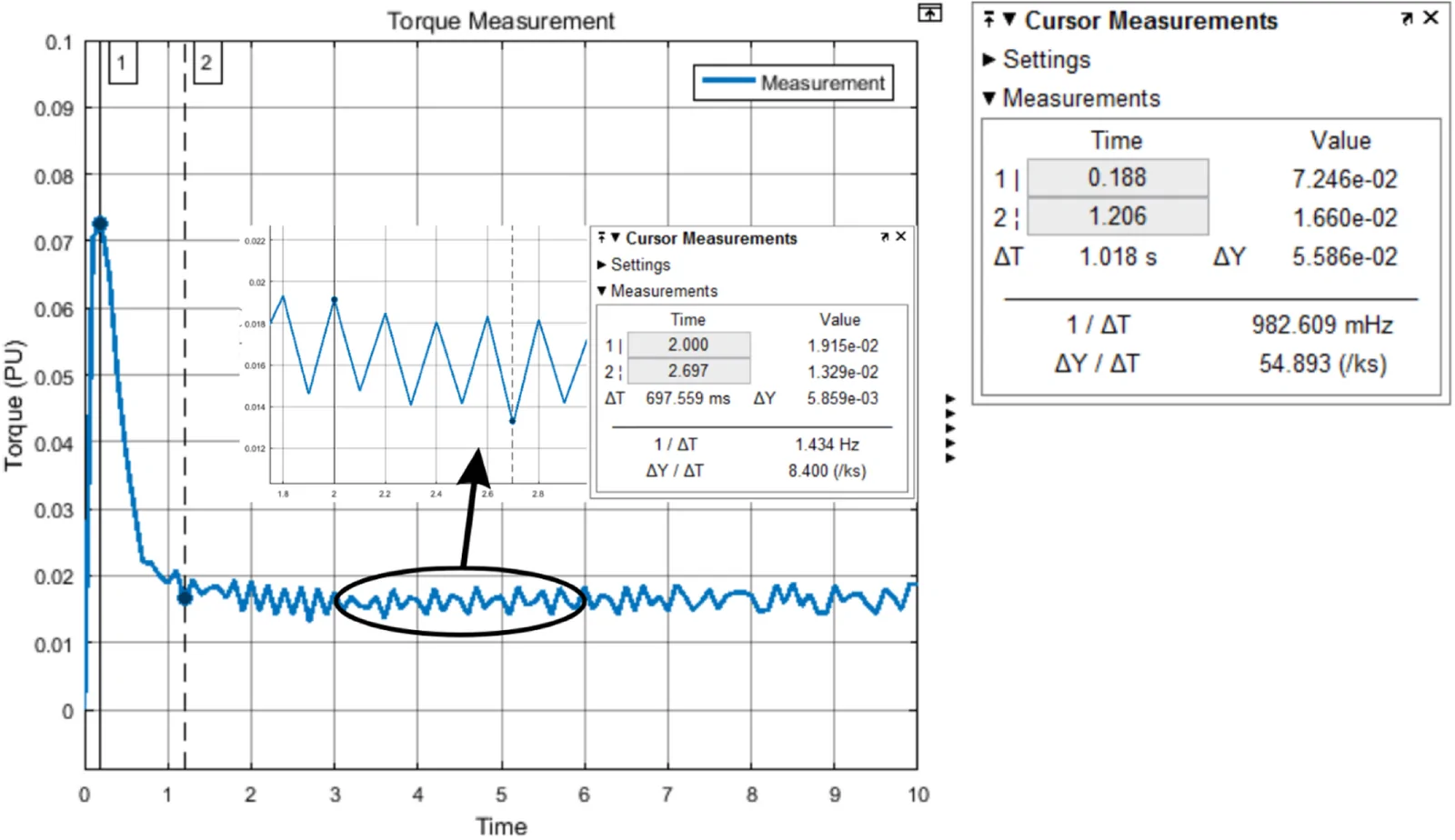
FOC-GWO torque response without load condition.

**Fig. 13:**
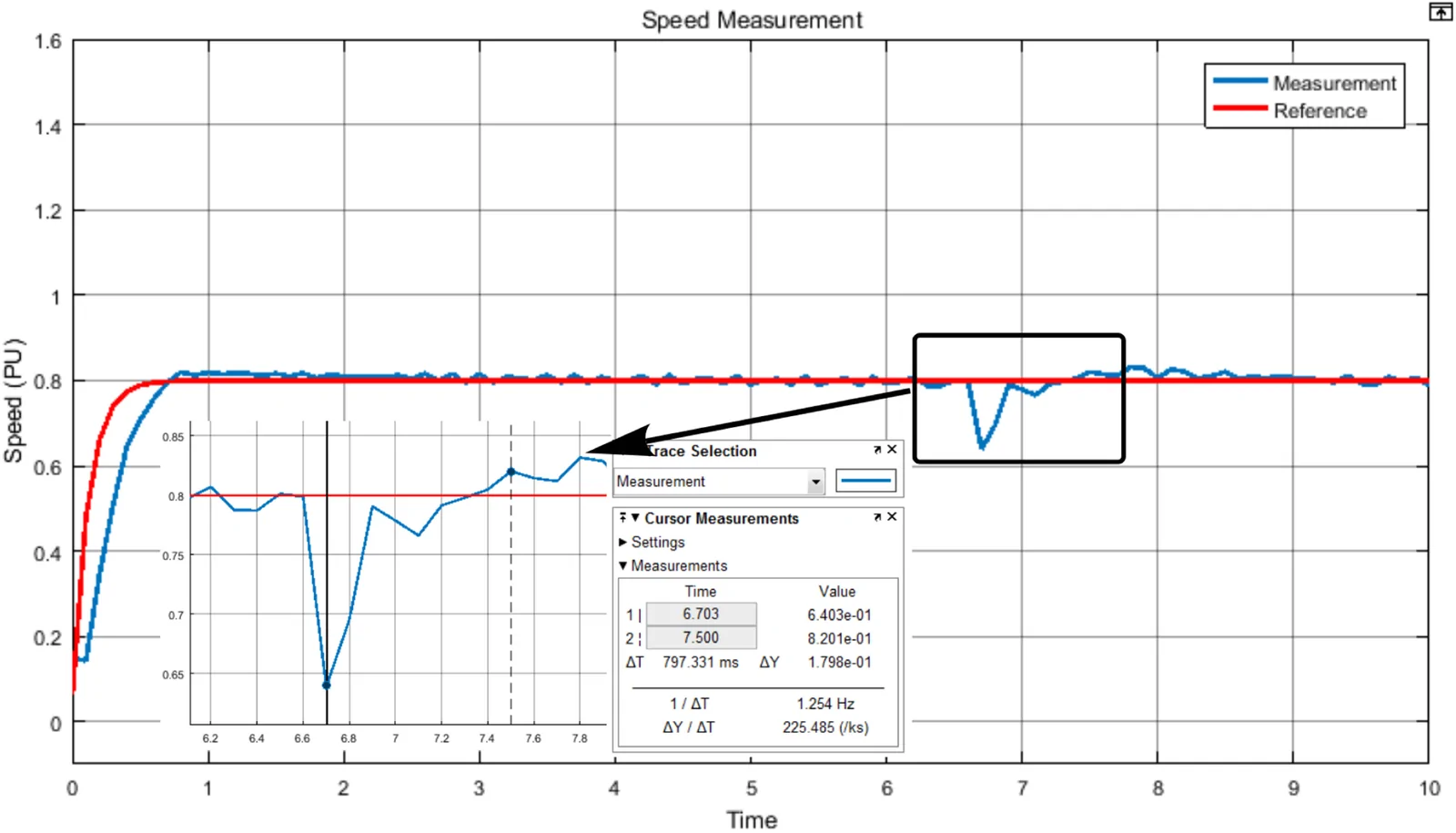
FOC+GWO speed response under load condition.

**Fig. 14:**
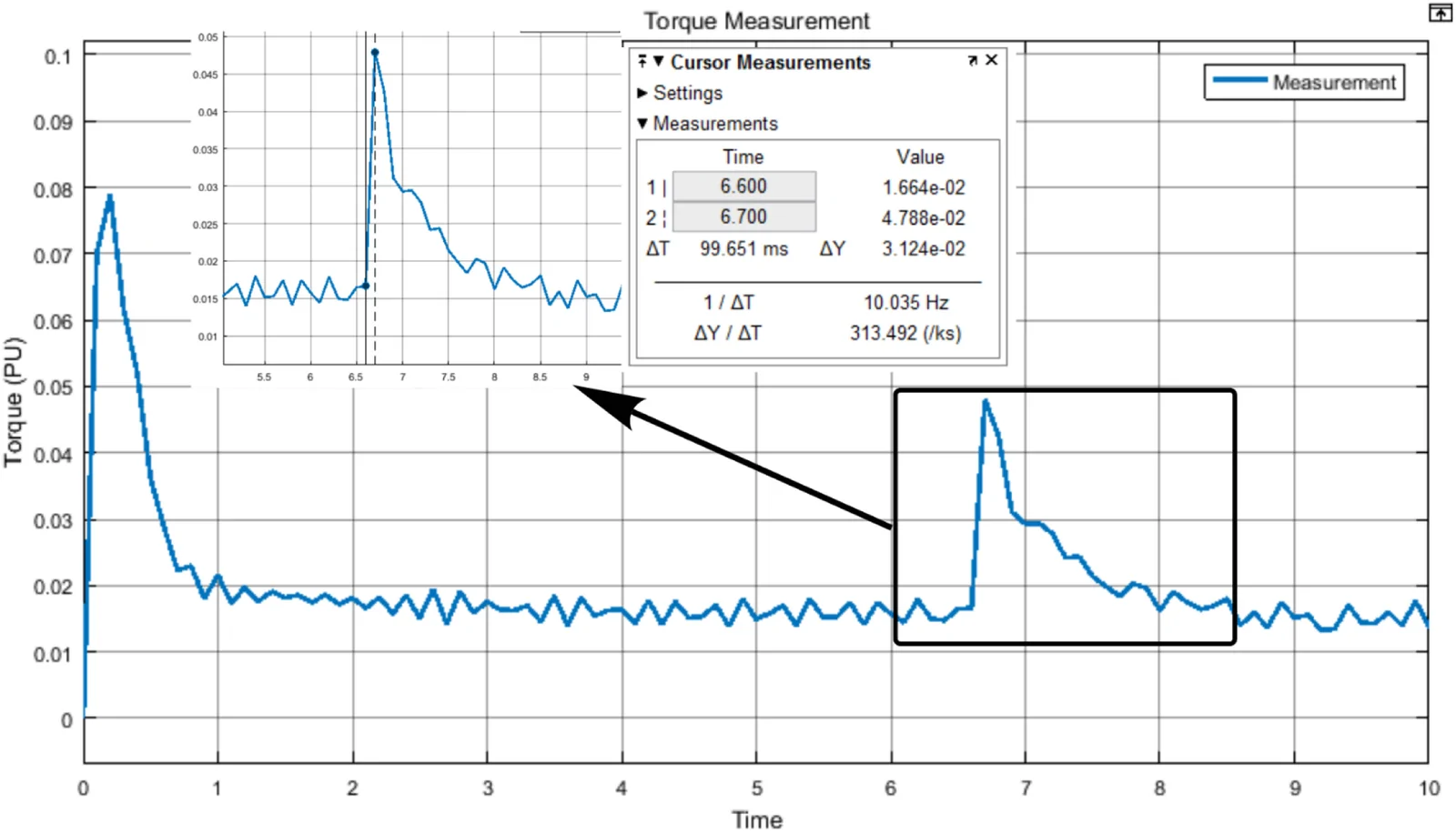
FOC+GWO torque response under load condition.

This section presents an analysis of the speed and torque responses of the FOC motor, with PI parameters optimized via GWO, under no-load conditions and under load disturbances, as shown in Fig. 11, Fig. 12, Fig. 13, Fig. 14.

### Response under no-load conditions

Test results show that the FOC method optimized using GWO can deliver good speed control performance under no-load conditions. Fig. 11. illustrates how the system achieves a rise time of approximately 0.5 s with relatively small overshoot (±3.75%), indicating a stable, not overly aggressive, initial response. Furthermore, the system reaches steady-state conditions in approximately 2 s with very small steady-state error. Under steady-state conditions, the resulting speed fluctuations are also very small, indicating that the FOC–GWO method effectively maintains the motor’s speed stability. The torque graph in Fig. 12. shows that FOC-GWO exhibits a more controlled torque response during startup than conventional FOC. The lower initial maximum torque value (±0.072 pu) indicates that the system generates sufficient acceleration without producing excessive torque spikes. The system’s transient response is smoother, with no significant undershoot, and it reaches steady state at approximately 1.2 s. In steady-state conditions, the torque ranges from 0.016 to 0.018 pu with relatively small ripple.

### Response under load disturbance conditions

The results of the FOC-GWO speed response test when a load disturbance is applied are shown in Fig. 13. The system’s stability characteristics improve compared to FOC without GWO optimization. During the startup phase, the system exhibits very little overshoot (±2.5%) and a stabilization time of approximately 1.0 s, indicating a more damped, stable response. When a sudden load disturbance was applied at approximately 6.7 s, the motor speed dropped to 0.64 pu, or about 20% of the reference value. However, the system recovered speed quickly within about 0.5 s, with a relatively small recovery overshoot (±3.75%). Additionally, the ripple in the steady-state condition was smaller than that in the conventional FOC method.

Fig. 14. shows how the response of the FOC-GWO produces smoother torque compared to conventional FOC control. During the startup phase, the initial torque surge is relatively small (±0.078 pu), indicating that control parameter optimization can reduce the effects of overly aggressive acceleration. Under steady-state conditions, the system maintains a stable torque with low ripple. When a sudden load disturbance is applied at approximately 6.7 s, the system responds by increasing the torque by ±0.047 pu to compensate for the additional load on the motor. After the disturbance, the system returns to a stable state within approximately 0.7 s, with relatively small oscillations.

Based on test results conducted under no-load conditions and with sudden load disturbances, the FOC method optimized using GWO (FOC-GWO) demonstrated better performance than conventional FOC in controlling the speed of a BLDC motor. Under no-load conditions, conventional FOC produces a relatively aggressive response with a speed overshoot of about 20%, whereas the FOC-GWO method can suppress overshoot to about 2–3%, resulting in a more damped transient response. Furthermore, the initial torque surge in conventional FOC reaches approximately 0.118 pu, whereas in FOC-GWO it is only about 0.072 pu, indicating that GWO-based optimization produces smoother, more stable motor acceleration characteristics.

The performance difference becomes more significant when the system experiences a sudden load disturbance. Under these conditions, the conventional FOC system experiences a greater speed drop and higher recovery oscillations before returning to a stable state. In contrast, the FOC-GWO method exhibits a smaller speed drop and a faster recovery process, accompanied by a lower surge in compensating torque. Additionally, under steady-state conditions, the FOC-GWO method exhibits lower speed and torque ripples than conventional FOC. Table 2 shows a quantitative comparison of conventional FOC and FOC-GWO.

The quantitative comparison in Table 3 clearly demonstrates the superiority of the proposed FOC–GWO method in terms of transient response, disturbance rejection, and steady-state stability.

**Table 3:** Performance comparison.

**Performance Metrics**	**Conventional FOC**	**FOC-GWO**	**Performance Analysis**
Speed Reference	0.8 pu	0.8 pu	Same
Rise Time (Speed)	0.30 s	0.45 s	FOC is slightly faster, but more aggressive
Peak Time (Speed)	0.50 s	0.85 s	FOC-GWO is more damped
Maximum Overshoot	20% (0.96 pu)	±3% (0.82 pu)	FOC-GWO is much more stable
Settling Time	1.2 s	1.0 s	FOC-GWO stabilizes slightly faster
Steady-State Error	0 pu	0 pu	Same
Speed Ripple (steady-state)	±0.01 pu	±0.005 pu	FOC-GWO is smoother
Torque Peak Start-up	0.118 pu	0.072 pu	FOC-GWO reduces torque spikes
Undershoot Torque	−0.007 pu	Not significant	FOC-GWO is more stable
Torque Ripple (steady-state)	±0.002 pu	±0.0015 pu	FOC-GWO is smaller
Speed Drop During Load Disturbance	Down to ±0.66 points	Down to approximately 0.65–0.70 points	FOC-GWO is more resistant to interference
Torque compensation during faults	0.07 pu	0.048 pu	FOC-GWO is more efficient
Recovery time after an outage	Longer	Faster	FOC-GWO is more responsive
Oscillations following a disturbance	Larger	Smaller	FOC-GWO is more stable

## Limitations

All experiments were conducted using the same load as a disturbance on the motor. This was done to ensure consistent test conditions, allowing differences in performance among the various motor control methods to be observed and compared more objectively.

The aspect of generality is supported at two different levels in this study. At the simulation/tuning level, the proposed framework is reconfigurable: the BLDC motor block in the Simulink FOC model is parameterized via scripts and direct settings on the Simulink block, so that specific motor parameters (e.g., pole pairs, stator resistance, stator inductance, rated torque, and rotor inertia) can be changed directly without altering the optimization algorithm or the tuning workflow itself. This means that the GWO-based tuning procedure, in principle can be re-run for other motor specifications simply by updating the parameters defined in the script and the motor block. However, at the hardware validation level, this framework has so far only been tested on a single physical BLDC motor platform (BLY171D) and has not yet been experimentally confirmed on other motor types. Expanding real-world hardware validation to additional motor platforms and a wider range of operating conditions is left for future research.

## Ethics statements

The authors confirm that no experiments involving human subjects or animals were conducted in connection with the present work, and that no data from social media platforms were used.

## CRediT author statement

**Rakhmat Agung Ramadhani:** Software, Formal Analysis, Writing-Original Draft, Resources, Data Curation, **Ika Noer Syamsiana:** Conceptualization, Methodology, Writing-Review & Editing, Supervision, **Arwin Datumaya Wahyudi Sumari:** Software, Writing-Review & Editing, Validation, Project Administration, Investigation, Validation.**Moechammad Sarosa:** Project Administration, Data Curation.

## Declaration of competing interest

The authors declare that they have no known competing financial interests or personal relationships that could have appeared to influence the work reported in this paper.

## Data Availability

Data will be made available on request.
